# P‐wave durations from automated electrocardiogram analysis to predict atrial fibrillation and mortality in heart failure

**DOI:** 10.1002/ehf2.14230

**Published:** 2022-12-02

**Authors:** Jiandong Zhou, Andrew Li, Martin Tan, Matthew Chung Yan Lam, Lok Tin Hung, Ronald Wing Hei Siu, Sharen Lee, Ishan Lakhani, Jeffrey Shi Kai Chan, Khalid Bin Waleed, Tong Liu, Kamalan Jeevaratnam, Qingpeng Zhang, Gary Tse

**Affiliations:** ^1^ School of Data Science City University of Hong Kong Hong Kong China; ^2^ Faculty of Science University of Calgary Calgary Canada; ^3^ University of Toronto Toronto Canada; ^4^ Li Ka Shing Institute of Health Sciences, Shenzhen Research Institute Chinese University of Hong Kong Shenzhen China; ^5^ Heart Failure and Structural Heart Disease Unit Cardiovascular Analytics Group, Hong Kong, China‐UK Collaboration Hong Kong China; ^6^ Department of Cardiology St George's Hospital NHS Foundation Trust London UK; ^7^ Tianjin Key Laboratory of Ionic‐Molecular Function of Cardiovascular Disease, Department of Cardiology, Tianjin Institute of Cardiology Second Hospital of Tianjin Medical University 300211 Tianjin China; ^8^ Faculty of Health and Medical Sciences University of Surrey GU2 7AL Guildford UK; ^9^ Kent and Medway Medical School University of Kent and Canterbury Christ Church University CT2 7NT Kent UK; ^10^ School of Nursing and Health Studies Hong Kong Metropolitan University Hong Kong China

**Keywords:** P‐wave duration, Inter‐atrial block, Heart failure, Stroke, Mortality

## Abstract

**Background:**

P‐wave indices have been used to predict incident atrial fibrillation (AF), stroke, and mortality. However, such indices derived from automated ECG measurements have not been explored for their predictive values in heart failure (HF). We investigated whether automated P‐wave indices can predict adverse outcomes in HF.

**Methods:**

This study included consecutive Chinese patients admitted to a single tertiary centre, presenting with HF but without prior AF, and with at least one baseline ECG, between 1 January 2010 and 31 December 2016, with last follow‐up of 31 December 2019.

**Results:**

A total of 2718 patients were included [median age: 77.4, interquartile range (IQR): (66.9–84.3) years; 47.9 males]. After a median follow‐up of 4.8 years (IQR: 1.9–9.0 years), 1150 patients developed AF (8.8/year), 339 developed stroke (2.6/year), 563 developed cardiovascular mortality (4.3/year), and 1972 had all‐cause mortality (15.1/year). Compared with 101–120 ms as a reference, maximum P‐wave durations predicted new‐onset AF at ≤90 ms [HR: 1.17(1.11, 1.50), *P* < 0.01], 131–140 ms [HR: 1.29(1.09, 1.54), *P* < 0.001], and ≥141 ms [HR: 1.52(1.32, 1.75), *P* < 0.001]. Similarly, they predicted cardiovascular mortality at ≤90 ms [HR: 1.50(1.08, 2.06), *P* < 0.001] or ≥141 ms [HR: 1.18(1.15, 1.45), *P* < 0.001], and all‐cause mortality at ≤90 ms [HR: 1.26(1.04, 1.51), *P* < 0.001], 131–140 ms [HR: 1.15(1.01, 1.32), *P* < 0.01], and ≥141 ms [HR: 1.31(1.18, 1.46), *P* < 0.001]. These remained significant after adjusting for significant demographics, past co‐morbidities, P‐wave dispersion, and maximum P‐wave amplitude.

**Conclusions:**

Extreme values of maximum P‐wave durations (≤90 ms and ≥141 ms) were significant predictors of new‐onset AF, cardiovascular mortality, and all‐cause mortality.

## Introduction

P‐wave duration (PWD) on the electrocardiogram (ECG) is a non‐invasive marker for intra‐atrial and inter‐atrial conduction times.^1^, ^2^ Prolonged PWDs, generally defined as PWD greater than 120 ms, reflecting inter‐atrial block (IAB) have been independently associated with adverse outcomes such as atrial fibrillation (AF) and stroke events in different disease cohorts,^3^, ^4^, ^5^, ^6^, ^7^ including in the general population.^8^ They have also been shown to predict AF recurrence after pulmonary vein isolation.^9^ By contrast, abnormally short PWDs have also been associated with adverse cardiac events. Short PWDs may reflect shorter atrial repolarization times and refractory periods, which would be expected to promote atrial arrhythmogenesis. Indeed, shorter minimum PWDs tended to be present in patients with paroxysmal lone AF, taking a median value of 60.5 ms.^10^ The Copenhagen ECG study found that PWDs less than 105 ms were an independent predictor of incident AF.^11^ Recently, it was found that short PWDs of less than 110 ms represented a marker of higher rate of AF recurrence after pulmonary vein isolation procedures.^12^

In heart failure (HF), there is pathophysiological remodelling of both the atria and ventricles.^13^, ^14^ Various P‐wave indices have been studied in terms of their ability to predict various adverse events in these settings. Partial IAB and an abnormal P‐wave terminal force in V1 (PTFV1) was a predictor of all‐cause mortality in HF patients with left ventricular ejection fraction less than 45.^15^ Advanced IAB was shown to predict new‐onset AF and ischaemic stroke in patients with HF.^16^ However, to date, there has been no study that specifically examined the use of P‐wave indices derived from automated ECG measurements for risk prediction in HF.

## Methods

### Study design and population

This study was approved by the Joint Chinese University of Hong Kong—New Territories East Cluster Clinical Research Ethics Committee. This was a retrospective cohort study of patients hospitalized for HF with ECG measurements recruited between 1 January 2010 and 31 December 2016 from a single tertiary centre in Hong Kong, China. Follow‐up was until 31 December 2019. The patients were identified from the Clinical Data Analysis and Reporting System (CDARS), a territory‐wide database that centralizes patient information from 43 local hospitals and their associated ambulatory and outpatient facilities to establish comprehensive medical data, including clinical characteristics, disease diagnosis, laboratory results, and drug treatment details. The system has been previously used by both our team and other teams in Hong Kong.^17^, ^18^, ^19^ Hospitalization for HF was identified by inpatient admissions with the principal diagnosis code of 428.X. Patients without 12‐lead ECG measurements and those with prior AF were excluded. Patients' demographics, prior co‐morbidities, hospitalization characteristics before and after initial ECG measurement date, medication prescriptions, laboratory examinations of complete blood counts, biochemical renal and liver function tests, and lipid and glucose tests were extracted. The *International Classification of Diseases, Ninth Revision, Clinical Modification* (ICD‐9‐CM) codes for comorbidities are detailed in *Table* S1. Automatically measured parameters from ECG related to the P‐wave, Q‐wave, R‐wave, S‐wave, and T‐wave were extracted. The baseline ECG obtained on the first HF admission was selected.

### Outcomes and statistical analysis

**Figure 1:**
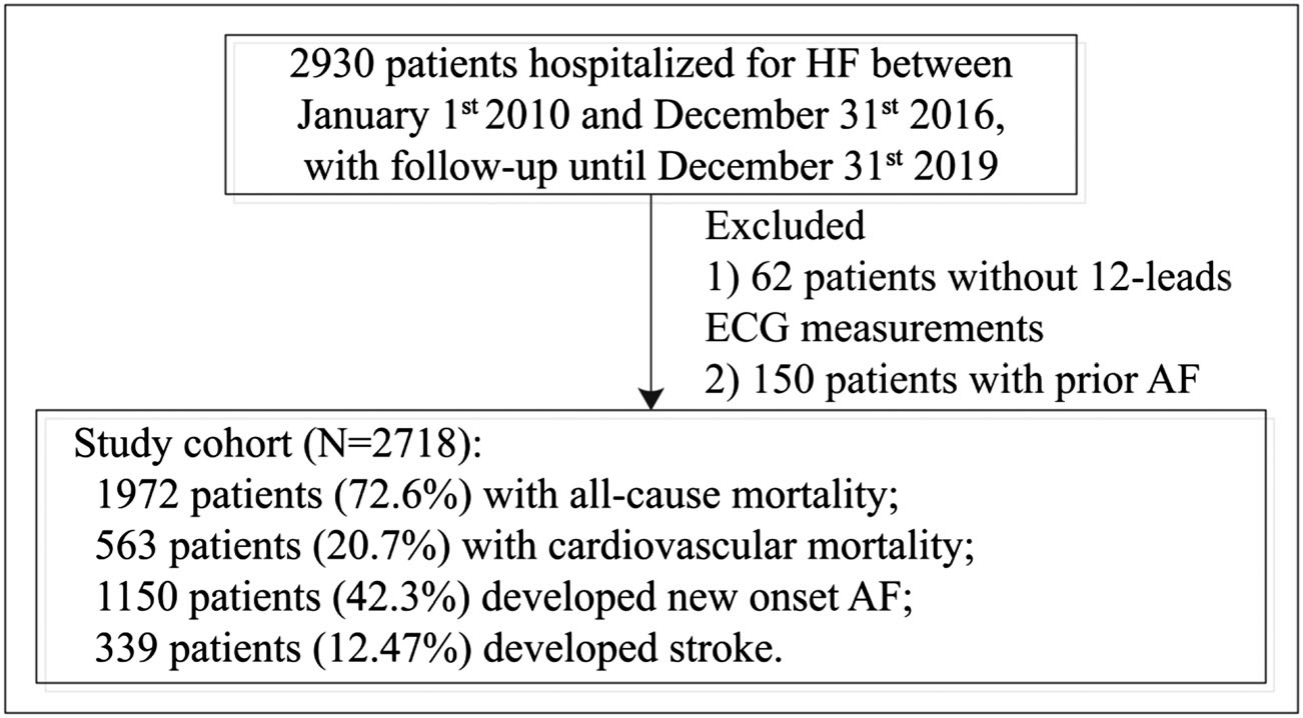
Procedures of data processing.

The primary outcome was new‐onset AF, and secondary outcomes include stroke, all‐cause mortality, and cardiovascular mortality, with follow‐up until 31 December 2019 (*Figure* *1*). Mortality data were obtained from the Hong Kong Death Registry, a population‐based official government registry with the registered death records of all Hong Kong citizens linked to CDARS. Likewise, data pertaining to new‐onset AF and stroke outcomes were also obtained from CDARS. Cardiovascular mortality was defined as mortality with the following ICD‐10 codes of I00‐I09, I11, I13, I20‐I51. There was no adjudication of the outcomes as this relied on the ICD‐9 coding or a record in the death registry. However, the coding was performed by the clinicians or administrative staff, who were not involved in this study.

Descriptive statistics were used to summarize baseline clinical characteristics of all patients with HF and based on the occurrence of the primary outcome. Continuous variables were presented as median [95% confidence interval (CI) or interquartile range (IQR)], and categorical variables were presented as count (%). The Mann–Whitney *U* test was used to compare continuous variables. The χ2 test with Yates' correction was used for 2 × 2 contingency data. Univariable Cox regression models were used to identify the significant risk factors of the primary and secondary outcomes. Hazard ratios (HRs) with corresponding 95% CIs and *P*‐values were reported. There was no imputation performed for missing data. No blinding was performed for the predictor as the values were obtained from the electronic health records automatically. All statistical tests were two‐tailed and considered significant if *P‐*value < 0.001. They were performed using RStudio software (Version 1.1.456) and Python (Version 3.6).

## Results

### Basic characteristics

**Figure 2:**
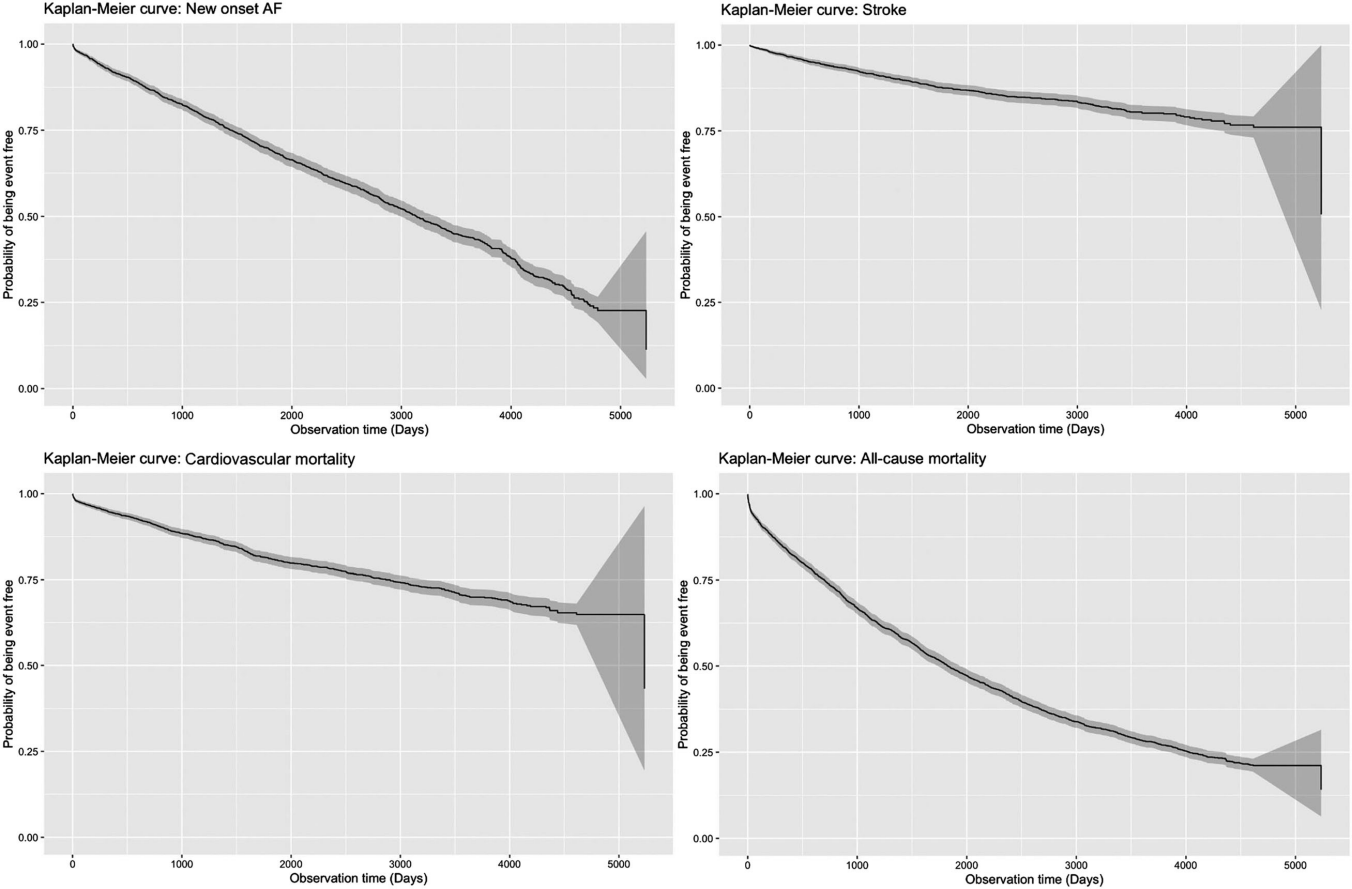
Kaplan–Meier curves of all‐cause mortality, cardiovascular mortality, new‐onset AF, and stroke.

**Figure 3:**
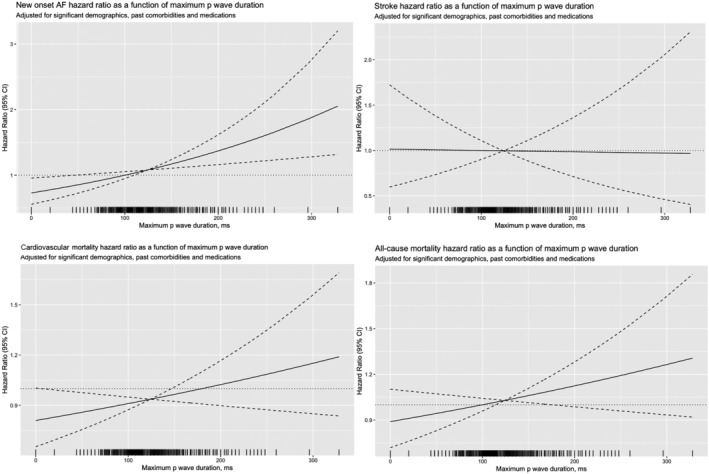
Adjusted cubic spline model (95% CI: dotted plot) of the associations between maximum P‐wave duration and the risks of adverse outcomes.

This study included 2718 HF patients without prior AF [median age: 77.4, IQR: (66.9–84.3) years; 47.9% males] with their main baseline characteristics summarized in *Table* *1*. The full list of variables analysed is shown in Table S2. Over a median follow‐up of 4.8 (1.9–9.0) years, 1150 patients developed AF, 339 developed stroke, 563 developed cardiovascular mortality, and 1972 had all‐cause mortality (*Figures* *2* and *3*). As seen from *Table* *1*, the incidence of AF was significantly higher among patients who were older at baseline and female. Moreover, subjects who developed AF were also more likely to eventually suffer from stroke (17.5% vs. 8.8%, *P* < 0.001) and cardiovascular mortality (24.3% vs. 18.0%; *P* = 0.0014) but not from all‐mortality (76.5% vs. 69.6%; *P* = 0.121) compared with those who remained in sinus rhythm throughout follow‐up.

**Table 1:** Baseline clinical characteristics of patients with/without new onset AF development

Characteristics	All (*N* = 2718) Median (IQR); count (%)	New‐onset AF (*N* = 1150) Median (IQR); count (%)	No new‐onset AF (*N* = 1568) Median (IQR); count (%)	*P* value^a^
Outcomes				
New‐onset AF	1150(42.31)	1150(100)	0(0)	<0.0001***
Stroke	339(12.47)	201(17.47)	138(8.80)	<0.0001***
Cardiovascular mortality	563(20.71)	280 (24.34)	283(18.04)	0.0014**
All‐cause mortality	1972(72.55)	880(76.52)	1092(69.64)	0.1213
Demographics				
Male gender	1302(47.90)	489(42.52)	813(51.84)	0.0046**
Baseline age, years	77.36(66.94–84.30)	77.88(68.37–84.47)	76.96(65.94–84.25)	0.0215*
<50	126(4.63)	32(2.78)	94(5.99)	0.0002***
50–60	257(9.45)	94(8.17)	163(10.39)	0.0863
60–70	449(16.51)	198(17.21)	251(16.00)	0.5104
70–80	775(28.51)	333(28.95)	442(28.18)	0.7754
>80	1111(40.87)	493(42.86)	618(39.41)	0.2563
Past co‐morbidities				
Charlson score	5.0(4.0–6.0)	5.0(4.0–6.0)	5.0(4.0–6.0)	0.3648
Diabetes without chronic complication	790(29.06)	315(27.39)	475(30.29)	0.2374
Diabetes with chronic complication	256(9.41)	65(5.65)	191(12.18)	<0.0001***
Hypertension	1253(46.10)	493(42.86)	760(48.46)	0.0839
Stroke/TIA	365(13.42)	186(16.17)	179(11.41)	0.0021**
Cancer	162(5.96)	58(5.04)	104(6.63)	0.1214
ECG measurements				
P‐wave front axis	55.0(34.0–72.0)	60.0(32.0–86.0)	54.0(35.0–68.0)	<0.0001***
P‐wave horizon axis	21.0(0.0–47.0)	32.0(4.0–82.5)	18.0(0.0–35.0)	<0.0001***
Heart rate	77.0(67.0–90.0)	79.0(68.0–93.0)	76.0(66.0–87.0)	<0.0001***
Mean PR interval	176.0(157.0–199.0)	180.0(160.0–204.0)	172.0(156.0–196.0)	<0.0001***
Mean QRS duration	91.0(82.0–108.0)	91.0(83.0–108.0)	91.0(82.0–109.5)	0.7348
Mean QTc	444.0(420.0–472.0)	446.0(423.0–473.0)	443.0(420.0–472.0)	0.1404
Mean P‐wave amplitude	0.04(0.01–0.06)	0.02(−0.0–0.05)	0.05(0.03–0.06)	<0.0001***
Max P‐wave amplitude	0.12(0.09–0.16)	0.11(0.08–0.14)	0.13(0.1–0.17)	<0.0001***
Min P‐wave amplitude	−0.09(−0.12 to −0.07)	−0.09(−0.12 to −0.07)	−0.1(−0.13 to −0.08)	<0.0001***
Max–min P‐wave amplitude	0.22(0.17–0.28)	0.2(0.15–0.26)	0.23(0.18–0.29)	<0.0001***
SD P‐wave amplitude	0.07(0.05–0.09)	0.06(0.05–0.08)	0.07(0.05–0.09)	<0.0001***
CV P‐wave amplitude	1.29(0.89–2.1)	1.23(−1.39–2.56)	1.3(1.01–1.93)	0.0002***
Mean P‐wave duration	78.83(67.58–87.79)	75.0(62.67–85.46)	81.0(71.83–89.46)	<0.0001***
Max P‐wave duration	120.0(108.0–136.0)	120.0(108.0–140.0)	119.0(108.0–136.0)	0.0118*
Min P‐wave duration	32.0(0.0–43.0)	28.0(0.0–40.0)	36.0(24.0–44.0)	<0.0001***
Max–min P‐wave duration	88.5(72.0–114.0)	96.0(76.0–121.0)	84.0(69.0–108.0)	<0.0001***
SD P‐wave duration	28.04(22.72–36.14)	30.0(23.66–39.02)	26.75(21.89–34.09)	<0.0001***
CV P‐wave duration	0.35(0.28–0.48)	0.4(0.3–0.6)	0.33(0.27–0.42)	<0.0001***
Mean P‐wave area	0.42(0.14–0.63)	0.23(−0.02–0.52)	0.5(0.3–0.7)	<0.0001***
Max P‐wave area	1.5(1.0–2.05)	1.3(0.9–1.9)	1.6(1.2–2.2)	<0.0001***
Min P‐wave area	−1.1(−1.5 to −0.8)	−1.0(−1.5 to −0.7)	−1.2(−1.6 to −0.8)	<0.0001***
Max–min P‐wave area	2.6(1.9–3.6)	2.4(1.7–3.3)	2.8(2.1–3.7)	<0.0001***
SD P‐wave area	0.75(0.56–0.99)	0.68(0.52–0.95)	0.8(0.6–1.03)	<0.0001***
CV P‐wave area	1.39(1.03–2.11)	1.32(−1.4–2.46)	1.41(1.12–1.98)	0.0001***
Mean p' wave amplitude	−0.01(−0.01–0.0)	−0.01(−0.01–0.0)	−0.01(−0.01–0.0)	0.0002***
Max p' wave amplitude	0.0(0.0–0.05)	0.01(0.0–0.05)	0.0(0.0–0.05)	<0.0001***
Min p' wave amplitude	−0.07(−0.09 to −0.05)	−0.06(−0.09 to −0.04)	−0.07(−0.09 to −0.05)	0.0127*
Max–min p' wave amplitude	0.08(0.06–0.12)	0.09(0.06–0.13)	0.08(0.06–0.12)	0.432
SD p' wave amplitude	0.03(0.03–0.04)	0.03(0.03–0.04)	0.03(0.03–0.04)	0.1466
CV p' wave amplitude	−1.99(−3.79 to −1.54)	−2.06(−3.84 to −1.44)	−1.97(−3.78 to −1.56)	0.6505
Mean p' wave duration	9.5(5.58–15.67)	10.0(6.0–16.17)	9.33(5.21–15.29)	0.0059**
Max p' wave duration	65.0(52.0–80.0)	70.0(56.0–84.0)	64.0(49.0–79.5)	<0.0001***
Min p' wave duration	‐	‐	‐	‐
Max–min p' wave duration	65.0(52.0–80.0)	70.0(56.0–84.0)	64.0(49.0–79.5)	<0.0001***
SD p' wave duration	28.63(24.21–34.02)	29.82(25.14–35.4)	27.98(23.56–33.06)	0.0007***
CV p' wave duration	1.66(1.4–1.88)	1.61(1.34–1.87)	1.72(1.48–1.88)	0.327
Mean P‐wave duration+ p' wave duration	88.17(75.21–98.96)	83.5(67.75–96.29)	90.5(80.58–100.21)	<0.0001***
Max P‐wave duration+ p' wave duration	128.0(112.0–151.5)	132.0(116.0–152.0)	125.0(112.0–148.0)	<0.0001***
Min P‐wave duration+ p' wave duration	44.0(0.0–60.0)	32.0(0.0–53.0)	48.0(30.5–62.0)	<0.0001***
Max–min P‐wave duration+ p' wave duration	90.0(65.0–120.0)	102.0(76.0–131.5)	80.0(60.0–112.0)	<0.0001***
SD P‐wave duration+ p' wave duration	26.91(19.43–37.11)	31.61(22.63–41.87)	23.82(17.83–33.13)	<0.0001***
CV P‐wave duration+ p' wave duration	0.3(0.21–0.46)	0.38(0.25–0.59)	0.26(0.2–0.37)	<0.0001***
Mean terminal P‐wave area	−0.04(−0.08 to −0.01)	−0.03(−0.08–0.0)	−0.04(−0.08 to −0.02)	0.0004***
Max terminal P‐wave area	0.0(0.0–0.3)	0.1(0.0–0.3)	0.0(0.0–0.2)	<0.0001***
Min terminal P‐wave area	−0.5(−0.8 to −0.2)	−0.4(−0.8 to −0.2)	−0.5(−0.7 to −0.3)	0.0736
Max–min terminal P‐wave area	0.6(0.3–1.0)	0.6(0.3–1.0)	0.6(0.3–0.9)	0.0363*
SD terminal P‐wave area	0.25(0.18–0.35)	0.26(0.18–0.38)	0.24(0.18–0.33)	0.0811
CV terminal P‐wave area	−2.18(−3.52 to −1.65)	−2.2(−3.85 to −1.5)	−2.18(−3.33 to −1.71)	0.8797

ACEI, angiotensin‐converting enzyme inhibitor; AF, atrial fibrillation; ARB, angiotensin II receptor blocker; COPD, chronic obstructive pulmonary disease; CV, coefficient of variation (mean/standard deviation); DPP‐4, dipeptidyl peptidase‐4 inhibitors; IHD, ischaemic heart disease; P′ wave, component of the P‐wave below the isoelectric line; PVD, peripheral vascular disease; SD, standard deviation; SGLT2, sodium‐glucose co‐transporter 2; TIA, transient ischaemic attack.

^a^ The comparisons were made between patients meeting primary new onset AF vs. those that did not.

* *P* ≤ 0.05.

** *P* ≤ 0.01.

*** *P* ≤ 0.001.

Significant differences were also found in relation to P‐wave indices. Specifically, subjects who experienced new‐onset AF also tended to have significantly larger maximum P‐wave durations (PWDs) across all leads (120 ms, IQR: 108–140 vs. 119 ms, IQR: 108–136, *P* = 0.01), and greater P‐wave dispersion, as measured by maximum–minimum difference (96 ms, IQR: 76.0–121.0 vs. 84 ms, IQR: 69.0–108.0; *P* < 0.0001), standard deviation (30, IQR: 23.66–39.02 vs. 26.75, IQR: 21.89–34.09; *P* < 0.0001), and coefficient of variation (0.4, IQR: 0.3–0.6 vs. 0.33, IQR: 0.27–0.42; *P* < 0.0001). By contrast, they had lower maximum P‐wave amplitude (0.02, IQR: −0.0‐0.05 vs. 0.05, IQR: 0.03–0.06; *P* < 0.0001) and lower maximum P‐wave area (1.3, IQR: 0.9–1.9 vs. 1.6, IQR: 1.2–2.2; *P* < 0.0001) relative to their normal counterparts.

PWD is the easiest parameter to obtain for the study of prognostic outcomes as it is usually part of the normal reporting of all ECGs in the clinical setting. As such, our subsequent analyses then summarized the cohort baseline characteristics based on maximum PWDs into <90 ms, 91–100 ms, 101–120 ms, 121–130 ms, 131–140 ms, and >140 ms (*Table* 2). The full list of variables analysed stratified by PWDs is shown in *Table* S3.

**Table 2:** Baseline clinical characteristics of study population stratified by maximum P wave duration * for *P* ≤ 0.05, ** for *P* ≤ 0.01, and *** for *P* ≤ 0.001

Characteristics	Overall (*N* = 2689) Median (IQR) or count (%)	<90 ms (*N* = 149) Median (IQR) or count (%)	91–100 ms (*N* = 272) Median (IQR) or count (%)	101–120 ms (*N* = 1041) Median (IQR) or count (%)	*P* value	121–130 ms (*N* = 355) Median (IQR) or count (%)	131–140 ms (*N* = 321) Median (IQR) or count (%)	>141 ms (*N* = 551) Median (IQR) or count (%)	*P* value
Outcomes									
New‐onset AF	1133(42.13%)	66(44.29%)	121(44.48%)	390(37.46%)	0.2751	152(42.81%)	147(45.79%)	257(46.64%)	0.779
Stroke	337(12.53%)	18(12.08%)	42(15.44%)	122(11.71%)	0.3486	56(15.77%)	39(12.14%)	60(10.88%)	0.1616
Cardiovascular mortality	553(20.56%)	40(26.84%)	57(20.95%)	211(20.26%)	0.3456	78(21.97%)	57(17.75%)	110(19.96%)	0.5346
Mortality	1953(72.62%)	119(79.86%)	185(68.01%)	724(69.54%)	0.5359	262(73.80%)	244(76.01%)	419(76.04%)	0.9534
Demographics									
Male gender	1297(48.23%)	77(51.67%)	130(47.79%)	541(51.96%)	0.7787	165(46.47%)	137(42.67%)	247(44.82%)	0.8278
Baseline age, years	77.37(66.93–84.3)	78.01(65.07–84.39); *n* = 149	74.66(63.06–82.32); *n* = 272	75.58(64.8–82.92); *n* = 1041	0.1912	76.69(65.7–83.41); *n* = 355	80.06(72.91–86.68); *n* = 321	80.18(71.57–86.9); *n* = 551	<0.0001***
<50	123(4.57%)	9(6.04%)	18(6.61%)	62(5.95%)	0.9295	16(4.50%)	5(1.55%)	13(2.35%)	0.0578
[50–60]	254(9.44%)	13(8.72%)	33(12.13%)	115(11.04%)	0.6286	37(10.42%)	21(6.54%)	35(6.35%)	0.0869
[60–70]	446(16.58%)	32(21.47%)	56(20.58%)	186(17.86%)	0.5326	68(19.15%)	35(10.90%)	69(12.52%)	0.0151*
[70–80]	765(28.44%)	31(20.80%)	73(26.83%)	312(29.97%)	0.1845	96(27.04%)	98(30.52%)	155(28.13%)	0.7468
>80	1101(40.94%)	64(42.95%)	92(33.82%)	366(35.15%)	0.4067	138(38.87%)	162(50.46%)	279(50.63%)	0.0761
Past co‐morbidities									
Charlson score	5.0(4.0–6.0)	5.0(3.0–7.0); *n* = 149	5.0(3.0–6.0); *n* = 272	5.0(4.0–6.0);n = 1,041	0.0796	5.0(4.0–7.0); *n* = 355	5.0(4.0–7.0); *n* = 321	5.0(4.0–7.0); *n* = 551	0.0047**
Diabetes without chronic complication	786(29.23%)	45(30.20%)	77(28.30%)	320(30.73%)	0.8491	100(28.16%)	92(28.66%)	152(27.58%)	0.9668
Diabetes with chronic complication	253(9.40%)	15(10.06%)	23(8.45%)	102(9.79%)	0.814	35(9.85%)	29(9.03%)	49(8.89%)	0.899
Hypertension	1245(46.29%)	64(42.95%)	111(40.80%)	467(44.86%)	0.7435	164(46.19%)	162(50.46%)	277(50.27%)	0.7408
Stroke/TIA	362(13.46%)	22(14.76%)	31(11.39%)	126(12.10%)	0.6597	50(14.08%)	45(14.01%)	88(15.97%)	0.7223
Cancer	162(6.02%)	11(7.38%)	10(3.67%)	63(6.05%)	0.2513	19(5.35%)	22(6.85%)	37(6.71%)	0.6853
ECG measurements									
Mean PR interval	161.42(142.58–181.83)	143.0(127.0–166.0); *n* = 149	162.0(148.5–176.0); *n* = 272	172.0(157.0–188.0); *n* = 1041	<0.0001***	183.0(166.5–203.0); *n* = 355	188.0(168.0–210.0); *n* = 321	196.0(168.0–228.0); *n* = 551	<0.0001***
Mean QRS duration	91.0(83.0–108.0)	97.0(83.0–132.0); *n* = 149	88.0(79.0–101.0); *n* = 272	91.0(82.0–106.0); *n* = 1041	<0.0001***	90.0(83.0–104.5); *n* = 355	91.0(83.0–108.0); *n* = 321	94.0(84.0–113.0); *n* = 551	0.1387
Mean QTc	444.0(420.0–472.0)	450.0(423.0–480.0); *n* = 149	436.0(414.0–463.0); *n* = 272	439.0(415.0–468.0); *n* = 1041	0.0074**	446.0(423.5–472.0); *n* = 355	450.0(429.0–476.0); *n* = 321	454.0(430.0–478.0); *n* = 551	0.0232*

ACEI, angiotensin‐converting enzyme inhibitor; AF, atrial fibrillation; ARB, angiotensin II receptor blocker; COPD, chronic obstructive pulmonary disease; CV, coefficient of variation (mean/standard deviation); DPP‐4, dipeptidyl peptidase‐4 inhibitors; IHD, ischaemic heart disease; P′ wave, component of the P‐wave below the isoelectric line; PVD, peripheral vascular disease; SD, standard deviation; SGLT2, sodium‐glucose co‐transporter 2; TIA, transient ischaemic attack.

* *P* ≤ 0.05.

** *P* ≤ 0.01.

*** *P* ≤ 0.001.

### Univariable and multivariable Cox regression for predicting adverse outcomes

Univariable Cox regression was conducted to identify significant risk factors for the different outcomes (*Table* *3*). For new‐onset AF, these were baseline age [HR: 1.04(1.04–1.05); *P* < 0.0001], Charlson score [HR: 1.18(1.15, 1.21); *P* < 0.0001], renal disease [HR: 1.26(1.04, 1.54); *P* = 0.02], systemic embolism [HR: 2.87(1.19, 6.91); *P* = 0.02], hypertension [HR: 1.22(1.08, 1.37); *P* = 0.0012], dementia and Alzheimer [HR: 2.76(1.24, 6.16); *P* = 0.0133], COPD [HR: 1.31(1.11, 1.56); *P* = 0.0017], peripheral vascular disease [HR: 1.65(1.13, 2.42); *P* = 0.01], prior stroke/TIA [HR: 1.69(1.45, 1.98); *P* < 0.0001], and gastrointestinal bleeding [HR: 1.56(1.33, 1.85); *P* < 0.0001].

**Table 3:** Univariable and multivariable adjusted risk of new‐onset AF, stroke, cardiovascular mortality, and all‐cause mortality based on max P‐wave duration

Model	Characteristics	New‐onset AF HR [CI]	Stroke HR [CI]	Cardiovascular mortality HR [CI]	All‐cause mortality HR [CI]
Model 1	≤90 ms	1.17[1.11, 1.50]**	1.11[0.69, 1.78]	1.50[1.08, 2.06]***	1.26[1.04, 1.51]***
	91–100 ms	0.89[0.74, 1.08]	1.14[0.83, 1.58]	0.91[0.69, 1.20]	0.83[0.71, 0.96]
	101–120 ms	1.00 [reference]	1.00 [reference]	1.00 [reference]	1.00 [reference]
	121–130 ms	0.98[0.83, 1.17]	1.29[0.97, 1.73]	1.06[0.83, 1.34]	1.01[0.89, 1.15]
	131–140 ms	1.29[1.09, 1.54]***	1.03[0.74, 1.44]	0.92[0.70, 1.21]	1.15[1.01, 1.32]**
	≥141 ms	1.52[1.32, 1.75]***	1.03[0.78, 1.37]	1.18[1.15, 1.45]***	1.31[1.18, 1.46]***
Model 2	≤90 ms	1.34[1.05, 1.72]***	1.18[0.73, 1.90]	1.62[1.17, 2.24]**	1.41[1.17, 1.69]***
	91–100 ms	0.98[0.81, 1.19]	1.24[0.89, 1.71]	1.00[0.76, 1.31]	0.97[0.84, 1.13]
	101–120 ms	1.00 [reference]	1.00 [reference]	1.00 [reference]	1.00 [reference]
	121–130 ms	1.04[0.88, 1.24]	1.34[1.00, 1.78]*	1.10[0.86, 1.40]	1.09[0.95, 1.24]
	131–140 ms	1.10[0.93, 1.31]	0.93[0.67, 1.30]	0.81[0.61, 1.07]	0.95[0.83, 1.09]
	≥141 ms	1.38[1.20, 1.59]***	0.96[0.73, 1.28]	1.08[0.88, 1.33]	1.15[1.03, 1.28]**
Model 3	≤90 ms	1.32[1.03, 1.70]***	1.17[0.73, 1.88]	1.61[1.16, 2.22]***	1.40[1.16, 1.69]***
	91–100 ms	0.99[0.82, 1.19]	1.25[0.90, 1.73]	1.00[0.76, 1.32]	0.99[0.85, 1.15]
	101–120 ms	1.00 [reference]	1.00 [reference]	1.00 [reference]	1.00 [reference]
	121–130 ms	1.04[0.87, 1.23]	1.33[1.00, 1.78]*	1.10[0.87, 1.40]	1.08[0.94, 1.23]
	131–140 ms	1.10[0.92, 1.31]***	0.93[0.66, 1.30]	0.81[0.61, 1.06]	0.94[0.82, 1.08]
	≥141 ms	1.37[1.19, 1.58]***	0.97[0.73, 1.28]	1.09[0.88, 1.34]	1.16[1.04, 1.29]**
Model 4	≤90 ms	1.78[1.38, 2.31]***	1.28[0.78, 2.09]	1.92[1.37, 2.69]***	1.68[1.38, 2.04]***
	91–100 ms	1.31[1.07, 1.60]**	1.37[0.97, 1.92]	1.14[0.85, 1.51]	1.11[0.95, 1.30]
	101–120 ms	1.00 [reference]	1.00 [reference]	1.00 [reference]	1.00 [reference]
	121–130 ms	1.11[0.93, 1.31]	1.35[1.01, 1.81]*	1.13[0.88, 1.43]	1.09[0.95, 1.24]
	131–140 ms	0.99[0.83, 1.18]	0.89[0.64, 1.26]	0.77[0.58, 1.01]	0.90[0.79, 1.03]
	≥141 ms	1.71[1.58, 1.86]***	0.74[0.51, 1.08]	1.76[1.57, 2.01]***	1.88[1.76, 2.02]**

Model 1: no adjustment. Model 2: adjusting for demographics. Model 3: adjusting for demographics and past co‐morbidities. Model 4: adjusting for demographics, past comorbidities, max–min P‐wave duration, maximum P‐wave amplitude. Adjustments were made for variables reaching *P* < 0.05 on univariable Cox regression.

* *P* ≤ 0.05.

** *P* ≤ 0.01.

*** *P* ≤ 0.001.

Patients were then stratified based on their maximum PWDs into ≤90 ms, 91–100 ms, 101–120 ms, 121–130 ms, 131–140 ms, and ≥ 141 ms. Compared with 101–120 ms as a reference, maximum PWDs ≤90 ms, 131–140 ms, and ≥141 ms were significant predictors of new‐onset AF [HR: 1.17(1.11, 1.50), *P* < 0.01, HR: 1.29(1.09, 1.54), *P* < 0.001 and HR: 1.52(1.32, 1.75), *P* < 0.001 respectively; model 1]. On multivariable analysis adjusting for significant demographics alone (model 2), with past co‐morbidities (model 3) or with past co‐morbidities, max–min P‐wave duration, maximum P‐wave amplitude (model 4), and maximum PWDs remained significant predictors (*Table* *3*). By contrast, maximum PWDs did not significantly predict stroke, except for 121–130 ms after multivariable adjustment (models 2, 3, and 4). Nevertheless, maximum PWDs predicted cardiovascular mortality at ≤90 ms [HR: 1.50(1.08, 2.06), *P* < 0.001] or ≥141 ms [HR: 1.18(1.15, 1.45), *P* < 0.001 for model 1] and after multivariable adjustment in models 2, 3, and 4. Finally, maximum PWDs predicted all‐cause mortality at ≤90 ms [HR: 1.26(1.04, 1.51), *P* < 0.001], 131–140 ms [HR: 1.15(1.01, 1.32), *P* < 0.01], and ≥141 ms [HR: 1.31(1.18, 1.46), *P* < 0.001].

## Discussion

The main findings of this study are that shorter (≤90 ms) and longer maximum PWDs (≥141 ms) were significantly associated with increased risks of new‐onset AF, cardiovascular mortality, and all‐cause mortality.

HF represents a global epidemic, imposing a significant burden on healthcare and economies worldwide. Several clinical parameters have been identified to aid the risk stratification of HF in attempts to improve patients' prognosis,^20^, ^21^, ^22^ with a particular focus on ECG variables. As it pertains to the ECG, different P‐wave indices have been associated with outcomes such as AF, stroke, and mortality.^23^, ^24^, ^25^, ^26^, ^27^ Moreover, PR interval, which reflects intra‐atrial, inter‐atrial, and atrioventricular conduction times, has been validated as an independent predictor of poor outcomes.^28^, ^29^ Subsequently, PWDs have been shown to be major contributor to the PR interval.^30^ Both shortening and prolongation in PWDs have been associated with adverse outcomes. Thus, short PWDs predicted higher AF recurrence rate after pulmonary vein isolation.^12^ By contrast, prolonged PWDs ≥120 ms predicted new‐onset AF and all‐cause mortality, whereas abnormal P‐wave terminal force in V1 predicted stroke.^31^ A study of HF patients receiving cardiac resynchronization therapy devices found that abnormal P‐wave terminal force in V1 and PWD ≥ 120 ms significantly predicted new‐onset AF and all‐cause mortality.^32^ Moreover, prolongations in amplified PWDs were predictive of new‐onset AF in patients with HF with preserved ejection fraction.^33^ Similar findings have been observed in the context of HF with reduced ejection fraction.^34^ These findings are in keeping with prolonged total atrial conduction time, which reflects atrial remodelling, with poorer cardiac prognosis in HF.^35^

Recent studies have explored the use of automated ECG indices for risk prediction in different cardiovascular diseases.^36^, ^37^ The Copenhagen ECG study found that PWDs less than 105 ms were an independent predictor of incident AF in the general population.^11^ A study from Japan found that prolonged PWDs derived from automatically assessed P‐waves were significant predictors of adverse cardiovascular events independently of left atrial enlargement in patients with at least one cardiovascular risk factor.^38^ The findings of our study suggest that PWDs at both extremes are predictive of poor outcomes, likely secondary to adverse atrial remodelling. Shortened PWDs reflect faster atrial repolarization that is associated with reduced refractoriness, whereas prolonged PWDs reflect conduction slowing and other conduction abnormalities, both representing re‐entrant substrates for arrhythmogenesis.^12^, ^39^, ^40^ The significance of these extremes in PWD, as illustrated in our study, has likewise been showcased in current literature, in that there appears to be a U‐wave correlation between PWD and HF risk with both low and high PWD values demonstrating significance.^41^ Although much of the existing data has focused on the detrimental influence of a prolonged PWD, further studies are still needed to evaluate the reasons as to why, beyond those suggested above, a shortened PWD is likewise associated with adverse cardiovascular prognosis.

It should be noted that the changes observed in ECG parameters secondary to left atrial dilation and remodelling in HF can likewise be applied to various other conditions, such as renal diseases. Variations in numerous variables, including but not limited to P‐wave duration, P‐wave dispersion, Tp‐e interval, and Tp‐e/QTc ratio, have all been shown in the setting of chronic kidney disease.^42^, ^43^ As such, albeit beyond the scope of the present study, it may be worth for future investigations to apply automated ECG measurements to assess the risk of developing AF and other arrhythmias in these conditions to further enhance risk stratification in the clinical setting.

## Limitations

There are limitations of this study that should be acknowledged. Firstly, it is based on a single centre cohort in Chinese patients, and therefore, our findings need to be validated in other ethnicities for greater generalizability. Secondly, this study was based on coded data from the central administrative database supplemented by automatically measured ECG variables. However, comprehensive medical records were not studied, and therefore, uncoded data, which include echocardiographic findings, were not included. Future studies should manually extract data from these domains to test whether their incorporation would improve risk prediction, as performed previously recently by us in a smaller HF cohort.^15^ Finally, ejection fraction data were not available for this cohort of patients, and as such, the variations in ejection fraction likely present among these patients could not be adjusted for in outcome analyses. It is widely known that a worsening ejection fraction itself is an independent predictor of a poorer prognosis in HF patients and in turn possibly contributed some degree of influence to the reported relationship between PWD and AF.

## Conclusions

Extreme values of maximum PWDs (≤90 ms and ≥141 ms) were significant predictors of new‐onset AF, cardiovascular mortality, and all‐cause mortality in HF.

## Conflict of interest

None.

## Author contributions

Jiandong Zhou and Andrew Li: Conception of study and literature search, preparation of figures, study design, data collection, data contribution, statistical analysis, data interpretation, manuscript drafting, and critical revision of manuscript. Martin Tan, Matthew Chung Yan Lam, Lok Tin Hung, Ronald Wing Hei Siu, Sharen Lee, Khalid Bin Waleed, Tong Liu, and Kamalan Jeevaratnam: Data collection, data analysis, statistical analysis, and critical revision of manuscript. Qingpeng Zhang and Gary Tse: Conception of study and literature search, study design, data collection, data analysis, data contribution, manuscript drafting, and critical revision of manuscript, study supervision.

## Supporting information


**Table S1.** ICD‐9 Codes for Comorbidities.
**Table S2.** Baseline clinical characteristics of patients with/without new onset AF development.
**Table S3.** Baseline clinical characteristics of study population stratified by maximum P wave duration.

## References

[1] Bayes de Luna A , Escobar‐Robledo LA , Aristizabal D , Weir Restrepo D , Mendieta G , Masso van Roessel A , Elosua R , Bayes‐Genis A , Martinez‐Selles M , Baranchuk A . Atypical advanced interatrial blocks: Definition and electrocardiographic recognition. J Electrocardiol. 2018 ‐ Dec; 51: 1091–1093. 30497736 10.1016/j.jelectrocard.2018.09.004

[2] Johner N , Namdar M , Shah DC . Intra‐ and interatrial conduction abnormalities: Hemodynamic and arrhythmic significance. J Interv Card Electrophysiol. 2018; 52: 293–302. 30128800 10.1007/s10840-018-0413-4

[3] Alexander B , Tse G , Martinez‐Selles M , Baranchuk A . Atrial conduction disorders. Curr Cardiol Rev. 2021; 17: 68–73. 33438553 10.2174/1573403X17666210112161524 PMC8142376

[4] Alexander B , Milden J , Hazim B , Haseeb S , Bayes‐Genis A , Elosua R , Martinez‐Selles M , Yeung C , Hopman W , Bayes de Luna A , Baranchuk A . New electrocardiographic score for the prediction of atrial fibrillation: The MVP ECG risk score (morphology‐voltage‐P‐wave duration). Ann Noninvasive Electrocardiol. 2019; 24: e12669. 31184409 10.1111/anec.12669 PMC6931412

[5] Chen LY , Soliman EZ . P wave indices‐Advancing our understanding of atrial fibrillation‐related cardiovascular outcomes. Front Cardiovasc Med. 2019; 6: 53. 31131284 10.3389/fcvm.2019.00053 PMC6509260

[6] Martinez‐Selles M , Elosua R , Ibarrola M , de Andres M , Diez‐Villanueva P , Bayes‐Genis A , Baranchuk A , Bayes‐de‐Luna A , Investigators BR . Advanced interatrial block and P‐wave duration are associated with atrial fibrillation and stroke in older adults with heart disease: The BAYES registry. Europace. 2020; 22: 1001–1008. 32449904 10.1093/europace/euaa114

[7] Alexander B , Haseeb S , van Rooy H , Tse G , Hopman W , Martinez‐Selles M , de Luna AB , Cinier G , Baranchuk A . Reduced P‐wave voltage in lead I is associated with development of atrial fibrillation in patients with coronary artery disease. J Atr Fibrillation. 2017; 10: 1657. 29487682 10.4022/jafib.1657 PMC5821633

[8] Maheshwari A , Norby FL , Soliman EZ , Alraies MC , Adabag S , O'Neal WT , Alonso A , Chen LY . Relation of prolonged P‐wave duration to risk of sudden cardiac death in the general population (from the atherosclerosis risk in communities study). Am J Cardiol. 2017; 119: 1302–1306. 28267962 10.1016/j.amjcard.2017.01.012 PMC5444665

[9] Caldwell J , Koppikar S , Barake W , Redfearn D , Michael K , Simpson C , Hopman W , Baranchuk A . Prolonged P‐wave duration is associated with atrial fibrillation recurrence after successful pulmonary vein isolation for paroxysmal atrial fibrillation. J Interv Card Electrophysiol. 2014; 39: 131–138. 24306110 10.1007/s10840-013-9851-1

[10] Chang IC , Austin E , Krishnan B , Benditt DG , Quay CN , Ling LH , Chen LY . Shorter minimum p‐wave duration is associated with paroxysmal lone atrial fibrillation. J Electrocardiol. 2014‐Feb; 47: 106–112. 24157188 10.1016/j.jelectrocard.2013.09.038

[11] Nielsen JB , Kuhl JT , Pietersen A , Graff C , Lind B , Struijk JJ , Olesen MS , Sinner MF , Bachmann TN , Haunso S , Nordestgaard BG , Ellinor PT , Svendsen JH , Kofoed KF , Kober L , Holst AG . P‐wave duration and the risk of atrial fibrillation: Results from the Copenhagen ECG study. Heart Rhythm. 2015; 12: 1887–1895. 25916567 10.1016/j.hrthm.2015.04.026

[12] Auricchio A , Ozkartal T , Salghetti F , Neumann L , Pezzuto S , Gharaviri A , Demarchi A , Caputo ML , Regoli F , De Asmundis C , Chierchia GB , Brugada P , Klersy C , Moccetti T , Schotten U , Conte G . Short P‐wave duration is a marker of higher rate of atrial fibrillation recurrences after pulmonary vein isolation: New insights into the pathophysiological mechanisms through computer simulations. J Am Heart Assoc. 2021: e018572. 33410337 10.1161/JAHA.120.018572 PMC7955300

[13] Senen K , Turhan H , Riza Erbay A , Basar N , Saatci Yasar A , Sahin O , Yetkin E . P‐wave duration and P‐wave dispersion in patients with dilated cardiomyopathy. Eur J Heart Fail. 2004; 6: 567–569. 15302004 10.1016/j.ejheart.2003.12.020

[14] Lakhani I , Leung KSK , Tse G , Lee APW . Novel mechanisms in heart failure with preserved, midrange, and reduced ejection fraction. Front Physiol. 2019; 10: 874. 31333505 10.3389/fphys.2019.00874 PMC6625157

[15] Tse G , Zhou J , Woo SWD , Ko CH , Lai RWC , Liu T , Liu Y , Leung KSK , Li A , Lee S , Li KHC , Lakhani I , Zhang Q . Multi‐modality machine learning approach for risk stratification in heart failure with left ventricular ejection fraction </= 45. ESC Heart Fail. 2020; 7: 3716–3725. 33094925 10.1002/ehf2.12929 PMC7754744

[16] Escobar‐Robledo LA , Bayes‐de‐Luna A , Lupon J , Baranchuk A , Moliner P , Martinez‐Selles M , Zamora E , de Antonio M , Domingo M , Cediel G , Nunez J , Santiago‐Vacas E , Bayes‐Genis A . Advanced interatrial block predicts new‐onset atrial fibrillation and ischemic stroke in patients with heart failure: The “Bayes' syndrome‐HF” study. Int J Cardiol. 2018; 271: 174–180. 29801761 10.1016/j.ijcard.2018.05.050

[17] Lee S , Liu T , Zhou J , Zhang Q , Wong WT , Tse G . Predictions of diabetes complications and mortality using hba1c variability: A 10‐year observational cohort study. Acta Diabetol. 2021; 58: 171–180. 32939583 10.1007/s00592-020-01605-6

[18] Ju C , Lai RWC , Li KHC , Hung JKF , Lai JCL , Ho J , Liu Y , Tsoi MF , Liu T , Cheung BMY , Wong ICK , Tam LS , Tse G . Comparative cardiovascular risk in users versus non‐users of xanthine oxidase inhibitors and febuxostat versus allopurinol users. Rheumatology (Oxford). 2020; 59: 2340–2349. 31873735 10.1093/rheumatology/kez576

[19] Zhou J , Wang X , Lee S , Wu WKK , Cheung BMY , Zhang Q , Tse G . Proton pump inhibitor or famotidine use and severe COVID‐19 disease: a propensity score‐matched territory‐wide study. Gut. 2021; 70: 2012–2013. 33277346 10.1136/gutjnl-2020-323668

[20] Chan JSK , Satti DI , Lee YHA , Hui JMH , Lee TTL , Chou OHI , Wai AKC , Ciobanu A , Liu Y , Liu T , Roever L , Biondi‐Zoccai G , Zhang Q , Cheung BMY , Zhou J , Tse G . High visit‐to‐visit cholesterol variability predicts heart failure and adverse cardiovascular events: A population‐based cohort study. Eur J Prev Cardiol. 2022; 29: e323–e325. 35653641 10.1093/eurjpc/zwac097

[21] Cheng Y , Shao Y , Weir CR , Shah RU , Bray BE , Garvin JH , Zeng‐Treitler Q . Predicting adverse outcomes in heart failure patients using different frailty status measures. Stud Health Technol Inform. 2017; 245: 327–331. 29295109 PMC5847261

[22] Bui AL , Horwich TB , Fonarow GC . Epidemiology and risk profile of heart failure. Nat Rev Cardiol. 2011; 8: 30–41. 21060326 10.1038/nrcardio.2010.165 PMC3033496

[23] Alonso A , Chen L . PR interval, P‐wave duration, and mortality: New insights, additional questions. Heart Rhythm. 2014; 11: 99–100. 24513921 10.1016/j.hrthm.2013.10.044

[24] Tse G , Lee S , Mok NS , Liu T , Chang D . Incidence and predictors of atrial fibrillation in a Chinese cohort of Brugada syndrome. Int J Cardiol. 2020; 314: 54–57. 32387420 10.1016/j.ijcard.2020.05.007

[25] Tse G , Wong CW , Gong M , Wong WT , Bazoukis G , Wong SH , Li G , Wu WKK , Tse LA , Lampropoulos K , Xia Y , Liu T , Baranchuk A , International Health Informatics Study N . Predictive value of inter‐atrial block for new onset or recurrent atrial fibrillation: A systematic review and meta‐analysis. Int J Cardiol. 2018; 250: 152–156. 29017777 10.1016/j.ijcard.2017.09.176

[26] He J , Tse G , Korantzopoulos P , Letsas KP , Ali‐Hasan‐Al‐Saegh S , Kamel H , Li G , Lip GYH , Liu T . P‐wave indices and risk of ischemic stroke: A systematic review and meta‐analysis. Stroke. 2017; 48: 2066–2072. 28679858 10.1161/STROKEAHA.117.017293

[27] Tse G , Lai ET , Yeo JM , Yan BP . Electrophysiological mechanisms of Bayes syndrome: Insights from clinical and mouse studies. Front Physiol. 2016; 7: 188. 27303306 10.3389/fphys.2016.00188 PMC4886053

[28] Schumacher K , Dagres N , Hindricks G , Husser D , Bollmann A , Kornej J . Characteristics of PR interval as predictor for atrial fibrillation: association with biomarkers and outcomes. Clin Res Cardiol. 2017; 106: 767–775. 28382425 10.1007/s00392-017-1109-y

[29] Nielsen JB , Pietersen A , Graff C , Lind B , Struijk JJ , Olesen MS , Haunso S , Gerds TA , Ellinor PT , Kober L , Svendsen JH , Holst AG . Risk of atrial fibrillation as a function of the electrocardiographic PR interval: Results from the Copenhagen ECG study. Heart Rhythm. 2013; 10: 1249–1256. 23608590 10.1016/j.hrthm.2013.04.012

[30] Soliman EZ , Cammarata M , Li Y . Explaining the inconsistent associations of PR interval with mortality: The role of P‐duration contribution to the length of PR interval. Heart Rhythm. 2014; 11: 93–98. 24096163 10.1016/j.hrthm.2013.10.003

[31] Tse G , Zhou J , Lee S , Liu Y , Leung KSK , Lai RWC , Burtman A , Wilson C , Liu T , Li KHC , Lakhani I , Zhang Q . Multi‐parametric system for risk stratification in mitral regurgitation: A multi‐task Gaussian prediction approach. Eur J Clin Invest. 2020; 50: e13321. 32535888 10.1111/eci.13321

[32] Jacobsson J , Carlson J , Reitan C , Borgquist R , Platonov PG . Interatrial block predicts atrial fibrillation and total mortality in patients with cardiac resynchronization therapy. Cardiology. 2020; 145: 720–729. 33022672 10.1159/000509916 PMC7677995

[33] Muller‐Edenborn B , Minners J , Kocher S , Chen J , Zeh W , Lehrmann H , Allgeier J , Neumann FJ , Arentz T , Jadidi A . Amplified P‐wave duration predicts new‐onset atrial fibrillation in patients with heart failure with preserved ejection fraction. Clin Res Cardiol. 2020; 109: 978–987. 31863175 10.1007/s00392-019-01590-z

[34] Abdellah AT , El‐Nagary M . Prevalence of P wave dispersion and interatrial block in patients with systolic heart failure and their relationship with functional status, hospitalization and one year mortality. Egypt Heart J. 2018; 70: 181–187. 30190644 10.1016/j.ehj.2018.02.006 PMC6123231

[35] Yamaura G , Watanabe T , Tamura H , Tsuchiya H , Hashimoto N , Wanezaki M , Nishiyama S , Arimoto T , Takahashi H , Yamauchi S , Shishido T , Yamanaka T , Miyamoto T , Watanabe M . Prolonged total atrial conduction time evaluated with tissue Doppler imaging predicts poor cardiac prognosis in patients with heart failure. Heart Vessels. 2019; 34: 1769–1776. 31028407 10.1007/s00380-019-01416-y

[36] Tse G , Lee S , Li A , Chang D , Li G , Zhou J , Liu T , Zhang Q . Automated electrocardiogram analysis identifies novel predictors of ventricular arrhythmias in Brugada syndrome. Front Cardiovasc Med. 2021; 7: 399. 10.3389/fcvm.2020.618254 PMC7840575 33521066

[37] Tse G , Lakhani I , Zhou J , Li KHC , Lee S , Liu Y , Leung KSK , Liu T , Baranchuk A , Zhang Q . P‐wave area predicts new onset atrial fibrillation in mitral stenosis: A machine learning approach. Front Bioeng Biotechnol. 2020; 8: 479. 32500070 10.3389/fbioe.2020.00479 PMC7243705

[38] Yokota A , Kabutoya T , Hoshide S , Kario K . Automatically assessed P‐wave predicts cardiac events independently of left atrial enlargement in patients with cardiovascular risks: The Japan morning surge home blood pressure study. J Clin Hypertens (Greenwich). 2020. 10.1111/jch.14136 PMC8029816 33340234

[39] Tse G , Lai ET , Lee AP , Yan BP , Wong SH . Electrophysiological mechanisms of gastrointestinal Arrhythmogenesis: Lessons from the heart. Front Physiol. 2016; 7: 230. 27378939 10.3389/fphys.2016.00230 PMC4906021

[40] Skibsbye L , Jespersen T , Christ T , Maleckar MM , van den Brink J , Tavi P , Koivumaki JT . Refractoriness in human atria: Time and voltage dependence of sodium channel availability. J Mol Cell Cardiol. 2016; 101: 26–34. 27773652 10.1016/j.yjmcc.2016.10.009

[41] Ostrowska B , Lind L , Sciaraffia E , Blomstrom‐Lundqvist C . Digitalised ECG measure of p‐wave duration predicts incident heart failure. EP Europace. 2022; 24: euac053.

[42] Kollu K , Altintepe L , Duran C , Topal M , Ecirli S . The assessment of P‐wave dispersion and myocardial repolarization parameters in patients with chronic kidney disease. Ren Fail. 2018; 40: 1–7. 29285964 10.1080/0886022X.2017.1419962 PMC6014377

[43] Szabo Z , Kakuk G , Fulop T , Matyus J , Balla J , Karpati I , Juhasz A , Kun C , Karanyi Z , Lorincz I . Effects of haemodialysis on maximum P wave duration and P wave dispersion. Nephrol Dial Transplant. 2002; 17: 1634–1638. 12198215 10.1093/ndt/17.9.1634

