# Supplementary material for: P‐wave durations from automated electrocardiogram analysis to predict atrial fibrillation and mortality in heart failure
Source: ESC Heart Fail. 2022 Dec 2;10(2):872–83. doi: 10.1002/ehf2.14230 (PMC10053164; doi:10.1002/ehf2.14230)
Supplement: Supplementary file 1 — Table S1. ICD‐9 Codes for Comorbidities. Table S2. Baseline clinical characteristics of patients with/without new onset AF development. Table S3. Baseline clinical characteristics of study population stratified by maximum P wave duration. [file EHF2-10-872-s001.docx]

**Supplementary Appendix**

**Supplementary Table 1. ICD-9 Codes for Comorbidities.**

| Diabetes without chronic complication 250 250.01 250.02 250.03 250.1 250.11 250.12 250.13 250.2 250.21 250.22 250.23 250.3 250.31 250.32 250.33 250.7 250.71 250.72 250.73 |
| --- |
| Diabetes with chronic complication 250.4 250.41 250.42 250.43 250.5 250.51 250.52 250.53 250.6 250.61 250.62 250.63 |
| Renal diseases 582 582 582.1 582.2 582.4 582.8 582.81 582.89 582.9 583 583 583.1 583.2 583.4 583.6 583.7 585 585.1 585.2 585.3 585.4 585.5 585.6 585.9 586 588 588 588.1 588.8 588.81 588.89 588.9 |
| Systemic embolism 444 444.01 444.09 444.1 444.2 444.21 444.22 444.8 444.81 444.89 444.9 445 445.01 445.02 445.8 445.81 445.89 |
| Hypertension 401 401.1 401.9 402 402.01 402.1 402.11 402.9 402.91 403 403.01 403.1 403.11 403.9 403.91 404 404.01 404.02 404.03 404.1 404.11 404.12 404.13 404.9 404.91 404.92 404.93 405 405.01 405.09 405.1 405.11 405.19 405.9 405.91 405.99 437.2 |
| Heart failure 428 428 428.1 428.2 428.2 428.21 428.22 428.23 428.3 428.3 428.31 428.32 428.33 428.4 428.4 428.41 428.42 428.43 428.9 398.91 402.01 402.11 402.91 404.01 404.03 404.11 404.13 404.91 404.93 |
| Atrial fibrillation 427.31 429.4 |
| Liver diseases 456 456.1 456.2 572.2 572.3 572.4 572.8 571.4 571.5 571.6 |
| Dementia and Alzheimer 331.82 290 290.1 290.11 290.12 290.13 290.2 290.21 290.3 290.4 290.41 290.42 290.43 290.8 290.9 294.2 294.1 294.11 294.21 332 46.1 333.4 340 42331 331.19 294.29 |
| COPD 490 491 492 493 494 495 496 491.1 491.2 491.21 491.22 491.8 491.9 492.8 493.01 493.02 493.1 493.11 493.12 493.2 493.21 493.22 493.8 493.81 493.82 493.9 493.91 493.92 494.1 495.1 495.2 495.3 495.4 495.5 495.6 495.7 495.8 495.9 |
| Peripheral vascular disease 250.7 443.9 443 443.1 443.2 443.21 443.22 443.23 443.24 443.29 443.8 443.81 443.82 443.89 441 443.9 785.4 V43.4 |
| Stroke/TIA Stroke/TIA 435 435.1 435.2 435.3 435.8 435.9 433.81 433.91 434 436 437 437.1 433.31 433.01 434.01 434.1 434.11 434.9 434.91 437.2 437.3 437.4 437.5 437.6 437.7 437.8 437.9 430 431 432 432.1 432.9 |
| Gastrointestinal bleeding 531 531.2 531.4 531.6 532 532.2 532.4 532.6 533 533.2 533.4 533.6 534 534.2 534.4 534.6 535.01 535.11 535.21 535.31 535.41 535.51 535.61 535.71 562.02 562.03 562.12 562.13 569.3 569.85 569.86 578 578.1 578.9 |
| IHD 410.01 410.02 410.1 410.11 410.12 410.2 410.21 410.22 410.3 410.31 410.32 410.4 410.41 410.42 410.5 410.51 410.52 410.6 410.61 410.62 410.7 410.71 410.72 410.8 410.81 410.82 410.9 410.91 410.92 411 411.1 411.8 411.81 411.89 413 413.1 413.9 414 414.01 414.02 414.03 414.04 414.05 414.06 414.07 414.1 414.11 414.12 414.19 414.2 414.3 414.4 414.8 414.9 410 412 |
| Cancer 140-239 |
| Obesity 278.01 278 278 |

**Supplementary Table 2. Baseline clinical characteristics of patients with/without new onset AF development.**

| **Characteristics** | **All (N=2718)**  **Median (IQR); Count (%)** | **New onset AF (N=1150)**  **Median (IQR); Count (%)** | **No new onset AF (N=1568)**  **Median (IQR); Count (%)** | **P value^#^** |
| --- | --- | --- | --- | --- |
| ***Outcomes*** |  |  |  |  |
| New onset AF | 1150(42.31) | 1150(100) | 0(0) | <0.0001*** |
| Stroke | 339(12.47) | 201(17.47) | 138(8.80) | <0.0001*** |
| Cardiovascular mortality | 563(20.71) | 280 (24.34) | 283(18.04) | 0.0014** |
| All-cause mortality | 1972(72.55) | 880(76.52) | 1092(69.64) | 0.1213 |
| ***Demographics*** |  |  |  |  |
| Male gender | 1302(47.90) | 489(42.52) | 813(51.84) | 0.0046** |
| Baseline age, years | 77.36(66.94-84.30) | 77.88(68.37-84.47) | 76.96(65.94-84.25) | 0.0215* |
| <50 | 126(4.63) | 32(2.78) | 94(5.99) | 0.0002*** |
| [50-60] | 257(9.45) | 94(8.17) | 163(10.39) | 0.0863 |
| [60-70] | 449(16.51) | 198(17.21) | 251(16.00) | 0.5104 |
| [70-80] | 775(28.51) | 333(28.95) | 442(28.18) | 0.7754 |
| >80 | 1111(40.87) | 493(42.86) | 618(39.41) | 0.2563 |
| ***Past comorbidities*** |  |  |  |  |
| Charlson score | 5.0(4.0-6.0 ) | 5.0(4.0-6.0) | 5.0(4.0-6.0) | 0.3648 |
| Diabetes without chronic complication | 790(29.06) | 315(27.39) | 475(30.29) | 0.2374 |
| Diabetes with chronic complication | 256(9.41) | 65(5.65) | 191(12.18) | <0.0001*** |
| Renal diseases | 345(12.69) | 110(9.56) | 235(14.98) | 0.0003*** |
| Systemic embolism | 7(0.25) | 5(0.43) | 2(0.12) | 0.2404 |
| Hypertension | 1253(46.10) | 493(42.86) | 760(48.46) | 0.0839 |
| Liver diseases | 15(0.55) | 3(0.26) | 12(0.76) | 0.1382 |
| Ventricular tachycardia/fibrillation | 135(4.96) | 34(2.95) | 101(6.44) | 0.0001*** |
| Dementia and Alzheimer | 14(0.51) | 6(0.52) | 8(0.51) | 0.8182 |
| COPD | 386(14.20) | 155(13.47) | 231(14.73) | 0.4547 |
| IHD | 1012(37.23) | 384(33.39) | 628(40.05) | 0.0177* |
| PVD | 66(2.42) | 27(2.34) | 39(2.48) | 0.919 |
| Stroke/TIA | 365(13.42) | 186(16.17) | 179(11.41) | 0.0021** |
| Gastrointestinal bleeding | 336(12.36) | 164(14.26) | 172(10.96) | 0.0270* |
| Cancer | 162(5.96) | 58(5.04) | 104(6.63) | 0.1214 |
| Obesity | 46(1.69) | 16(1.39) | 30(1.91) | 0.3818 |
| ***Medications*** |  |  |  |  |
| ACEI | 1259(46.32) | 530(46.08) | 729(46.49) | 0.9264 |
| ARB | 257(9.45) | 130(11.30) | 127(8.09) | 0.0126* |
| Calcium channel blockers | 1232(45.32) | 528(45.91) | 704(44.89) | 0.7739 |
| Beta blockers | 1334(49.08) | 597(51.91) | 737(47.00) | 0.1495 |
| Diuretics for hypertension | 319(11.73) | 130(11.30) | 189(12.05) | 0.636 |
| Diuretics for heart failure | 1092(40.17) | 525(45.65) | 567(36.16) | 0.0013** |
| Nitrates | 749(27.55) | 318(27.65) | 431(27.48) | 0.9762 |
| Antihypertensive drugs | 511(18.80) | 204(17.73) | 307(19.57) | 0.3395 |
| Statins and fibrates | 829(30.50) | 294(25.56) | 535(34.11) | 0.0005*** |
| Lipid-lowering drugs | 648(23.84) | 230(20.00) | 418(26.65) | 0.0018** |
| Anticoagulants | 503(18.50) | 370(32.17) | 133(8.48) | <0.0001*** |
| Antiplatelets | 1117(41.09) | 487(42.34) | 630(40.17) | 0.4858 |
| Sodium-glucose cotransporter 2 inhibitors | 44(1.61) | 19(1.65) | 25(1.59) | 0.9699 |
| Dipeptidyl peptidase-4 inhibitors | 207(7.61) | 79(6.86) | 128(8.16) | 0.2748 |
| Alpha-glucosidase inhibitors | 69(2.53) | 29(2.52) | 40(2.55) | 0.9389 |
| Proton pump inhibitors | - | - | - | - |
| ***Complete blood counts*** |  |  |  |  |
| Haemoglobin, g/dL | 11.8(10.4-13.3) | 12.0(10.7-13.5) | 11.7(10.1-13.1) | 0.0003*** |
| Mean corpuscular volume, fL | 92.4(87.9-96.0) | 92.7(88.3-96.4) | 91.9(87.6-95.8) | 0.0509 |
| Basophil, x10^9/L | 0.0(0.0-0.03) | 0.0(0.0-0.02) | 0.0(0.0-0.03) | 0.3442 |
| Eosinophil, x10^9/L | 0.1(0.09-0.2) | 0.1(0.04-0.2) | 0.12(0.1-0.3) | <0.0001*** |
| Lymphocyte, x10^9/L | 1.2(0.8-1.69) | 1.2(0.8-1.6) | 1.2(0.8-1.7) | 0.1119 |
| Blast, x10^9/L | 0.0(0.0-0.1 | 0.0(0.0-0.1) | 0.0(0.0-0.1) | 0.278 |
| Metamyelocyte, x10^9/L | 0.12(0.06-0.22) | 0.13(0.08-0.22) | 0.12(0.05-0.2) | 0.416 |
| Monocyte, x10^9/L | 0.5(0.4-0.7) | 0.55(0.4-0.7) | 0.5(0.4-0.7) | 0.5275 |
| Neutrophil, x10^9/L | 5.0(3.8-7.0 | 4.89(3.7-6.8) | 5.13(3.9-7.1) | 0.0386* |
| White cell count, x10^9/L | 7.2(5.8-9.0) | 7.0(5.5-8.6) | 7.32(6.0-9.2) | 0.0029** |
| Mean cell haemoglobin, pg | 31.4(29.6-32.9 | 31.5(29.7-32.9) | 31.4(29.6-32.85) | 0.6358 |
| Myelocyte, x10^9/L | 0.08(0.04-0.2 | 0.08(0.04-0.38) | 0.09(0.04-0.18) | 0.558 |
| Platelet, x10^9/L | 195.5(153.0-239.0 | 181.0(144.0-228.0) | 206.5(166.0-250.0) | <0.0001*** |
| Reticulocyte, x10^9/L | 42.25(29.26-59.03 | 32.4(25.15-46.85) | 50.8(31.06-64.78) | 0.339 |
| Red blood count, x10^12/L | 3.89(3.36-4.38) | 3.97(3.43-4.43) | 3.84(3.32-4.34) | 0.0024** |
| Hematocrit, L/L | 0.38(0.32-0.4) | 0.35(0.32-0.39) | 0.39(0.33-0.4) | 0.2129 |
| ***Liver and renal functions*** |  |  |  |  |
| Potassium, mmol/L | 4.2(3.8-4.6) | 4.1(3.8-4.5) | 4.2(3.9-4.6) | 0.0014** |
| Urate, mmol/L | 0.46(0.38-0.56) | 0.47(0.37-0.57) | 0.46(0.38-0.56) | 0.7994 |
| Albumin, g/L | 39.6(35.9-42.7) | 39.7(36.3-42.6) | 39.6(35.55-42.7) | 0.3627 |
| Sodium, mmol/L | 140.5(138.2-142.4) | 140.7(138.3-142.5) | 140.4(138.1-142.4) | 0.4355 |
| Urea, mmol/L | 7.8(5.9-12.02) | 7.54(5.9-10.86) | 8.11(5.9-13.2) | 0.0345* |
| Protein, g/L | 72.6(68.0-76.4) | 72.9(68.54-76.65) | 72.4(67.8-76.0) | 0.0198* |
| Creatinine, umol/L | 109.0(85.0-153.0) | 107.0(84.0-140.0) | 112.0(85.0-172.0) | 0.005** |
| Alkaline phosphatase, U/L | 77.0(62.0-99.0) | 79.0(64.0-100.0) | 76.0(62.0-99.0) | 0.2213 |
| Aspartate transaminase, U/L | 26.5(20.0-38.0) | 27.5(22.5-37.5) | 26.0(16.5-39.0) | 0.3062 |
| Alanine transaminase, U/L | 19.0(14.0-27.0) | 19.0(14.0-26.5) | 19.0(14.0-28.0) | 0.1463 |
| Bilirubin, umol/L | 10.6(7.6-15.0) | 12.4(9.0-17.5) | 9.0(7.0-13.0) | <0.0001*** |
| ***Lipid and glucose tests*** |  |  |  |  |
| Triglyceride, mmol/L | 1.19(0.88-1.74) | 1.11(0.84-1.69) | 1.24(0.9-1.8) | 0.0125* |
| Low-density lipoprotein, mmol/L | 2.28(1.8-2.9) | 2.26(1.73-2.89) | 2.31(1.84-2.9) | 0.1986 |
| High-density lipoprotein, mmol/L | 1.25(1.0-1.54) | 1.24(1.0-1.5) | 1.25(1.0-1.57) | 0.201 |
| Cholesterol, mmol/L | 4.3(3.6-5.0) | 4.2(3.5-4.9) | 4.3(3.66-5.04) | 0.0191* |
| Glucose, mmol/L | 6.3(5.4-8.2) | 6.37(5.43-8.14) | 6.2(5.37-8.3) | 0.4382 |
| ***Cardiac, clotting, inflammatory,***  ***and acid-base tests*** |  |  |  |  |
| D-dimer, ng/mL | 1315.21(750.33-2692.78) | 1265.76(686.02-2608.61) | 1380.58(823.64-2879.63) | 0.2199 |
| High sensitive troponin-I, ng/L | 0.03(0.02-0.07) | 0.03(0.02-0.06) | 0.03(0.02-0.08) | 0.1841 |
| Lactate dehydrogenase, U/L | 237.0(194.0-303.5) | 241.0(198.0-312.0) | 234.0(192.0-299.0) | 0.0077** |
| APTT, second | 33.4(29.9-39.75) | 36.5(31.5-42.2) | 31.3(29.0-35.0) | <0.0001*** |
| Prothrombin time/INR, second | 12.4(10.9-19.85) | 16.5(11.7-23.35) | 11.3(10.4-12.7) | <0.0001*** |
| ***ECG measurements*** |  |  |  |  |
| P wave front axis | 55.0(34.0-72.0) | 60.0(32.0-86.0) | 54.0(35.0-68.0) | <0.0001*** |
| P wave horizon axis | 21.0(0.0-47.0 | 32.0(4.0-82.5) | 18.0(0.0-35.0) | <0.0001*** |
| Heart rate | 77.0(67.0-90.0) | 79.0(68.0-93.0) | 76.0(66.0-87.0) | <0.0001*** |
| Mean PR interval | 176.0(157.0-199.0) | 180.0(160.0-204.0) | 172.0(156.0-196.0) | <0.0001*** |
| Mean QRS duration | 91.0(82.0-108.0) | 91.0(83.0-108.0) | 91.0(82.0-109.5) | 0.7348 |
| Mean QTc | 444.0(420.0-472.0) | 446.0(423.0-473.0) | 443.0(420.0-472.0) | 0.1404 |
| Mean P wave amplitude | 0.04(0.01-0.06) | 0.02(-0.0-0.05) | 0.05(0.03-0.06) | <0.0001*** |
| Max P wave amplitude | 0.12(0.09-0.16) | 0.11(0.08-0.14) | 0.13(0.1-0.17) | <0.0001*** |
| Min P wave amplitude | -0.09(-0.12 - -0.07) | -0.09(-0.12 - -0.07) | -0.1(-0.13 - -0.08) | <0.0001*** |
| Max-min P wave amplitude | 0.22(0.17-0.28) | 0.2(0.15-0.26) | 0.23(0.18-0.29) | <0.0001*** |
| SD P wave amplitude | 0.07(0.05-0.09) | 0.06(0.05-0.08) | 0.07(0.05-0.09) | <0.0001*** |
| CV P wave amplitude | 1.29(0.89-2.1) | 1.23(-1.39-2.56) | 1.3(1.01-1.93) | 0.0002*** |
| Mean P wave duration | 78.83(67.58-87.79) | 75.0(62.67-85.46) | 81.0(71.83-89.46) | <0.0001*** |
| Max P wave duration | 120.0(108.0-136.0) | 120.0(108.0-140.0) | 119.0(108.0-136.0) | 0.0118* |
| Min P wave duration | 32.0(0.0-43.0) | 28.0(0.0-40.0 | 36.0(24.0-44.0) | <0.0001*** |
| Max-min P wave duration | 88.5(72.0-114.0) | 96.0(76.0-121.0) | 84.0(69.0-108.0) | <0.0001*** |
| SD P wave duration | 28.04(22.72-36.14) | 30.0(23.66-39.02) | 26.75(21.89-34.09) | <0.0001*** |
| CV P wave duration | 0.35(0.28-0.48) | 0.4(0.3-0.6) | 0.33(0.27-0.42) | <0.0001*** |
| Mean P wave area | 0.42(0.14-0.63) | 0.23(-0.02-0.52) | 0.5(0.3-0.7) | <0.0001*** |
| Max P wave area | 1.5(1.0-2.05) | 1.3(0.9-1.9) | 1.6(1.2-2.2) | <0.0001*** |
| Min P wave area | -1.1(-1.5 - -0.8) | -1.0(-1.5 - -0.7) | -1.2(-1.6 - -0.8) | <0.0001*** |
| Max-min P wave area | 2.6(1.9-3.6) | 2.4(1.7-3.3) | 2.8(2.1-3.7) | <0.0001*** |
| SD P wave area | 0.75(0.56-0.99) | 0.68(0.52-0.95) | 0.8(0.6-1.03) | <0.0001*** |
| CV P wave area | 1.39(1.03-2.11) | 1.32(-1.4-2.46) | 1.41(1.12-1.98) | 0.0001*** |
| Mean p' wave amplitude | -0.01(-0.01-0.0) | -0.01(-0.01-0.0) | -0.01(-0.01-0.0) | 0.0002*** |
| Max p' wave amplitude | 0.0(0.0-0.05) | 0.01(0.0-0.05) | 0.0(0.0-0.05) | <0.0001*** |
| Min p' wave amplitude | -0.07(-0.09- -0.05) | -0.06(-0.09 - -0.04) | -0.07(-0.09 - -0.05) | 0.0127* |
| Max-min p' wave amplitude | 0.08(0.06-0.12) | 0.09(0.06-0.13) | 0.08(0.06-0.12) | 0.432 |
| SD p' wave amplitude | 0.03(0.03-0.04) | 0.03(0.03-0.04) | 0.03(0.03-0.04) | 0.1466 |
| CV p' wave amplitude | -1.99(-3.79- -1.54) | -2.06(-3.84 - -1.44) | -1.97(-3.78 - -1.56) | 0.6505 |
| Mean p' wave duration | 9.5(5.58-15.67) | 10.0(6.0-16.17) | 9.33(5.21-15.29) | 0.0059** |
| Max p' wave duration | 65.0(52.0-80.0) | 70.0(56.0-84.0) | 64.0(49.0-79.5) | <0.0001*** |
| Min p' wave duration | - | - | - | - |
| Max-min p' wave duration | 65.0(52.0-80.0) | 70.0(56.0-84.0) | 64.0(49.0-79.5) | <0.0001*** |
| SD p' wave duration | 28.63(24.21-34.02) | 29.82(25.14-35.4) | 27.98(23.56-33.06) | 0.0007*** |
| CV p' wave duration | 1.66(1.4-1.88) | 1.61(1.34-1.87) | 1.72(1.48-1.88) | 0.327 |
| Mean P wave duration+ p' wave duration | 88.17(75.21-98.96) | 83.5(67.75-96.29) | 90.5(80.58-100.21) | <0.0001*** |
| Max P wave duration+ p' wave duration | 128.0(112.0-151.5) | 132.0(116.0-152.0) | 125.0(112.0-148.0) | <0.0001*** |
| Min P wave duration+ p' wave duration | 44.0(0.0-60.0) | 32.0(0.0-53.0) | 48.0(30.5-62.0) | <0.0001*** |
| Max-min P wave duration+ p' wave duration | 90.0(65.0-120.0) | 102.0(76.0-131.5) | 80.0(60.0-112.0) | <0.0001*** |
| SD P wave duration+ p' wave duration | 26.91(19.43-37.11) | 31.61(22.63-41.87) | 23.82(17.83-33.13) | <0.0001*** |
| CV P wave duration+ p' wave duration | 0.3(0.21-0.46) | 0.38(0.25-0.59) | 0.26(0.2-0.37) | <0.0001*** |
| Mean terminal P wave area | -0.04(-0.08- -0.01) | -0.03(-0.08-0.0) | -0.04(-0.08 - -0.02) | 0.0004*** |
| Max terminal P wave area | 0.0(0.0-0.3) | 0.1(0.0-0.3) | 0.0(0.0-0.2) | <0.0001*** |
| Min terminal P wave area | -0.5(-0.8- -0.2) | -0.4(-0.8 - -0.2) | -0.5(-0.7 - -0.3) | 0.0736 |
| Max-min terminal P wave area | 0.6(0.3-1.0) | 0.6(0.3-1.0) | 0.6(0.3-0.9) | 0.0363* |
| SD terminal P wave area | 0.25(0.18-0.35) | 0.26(0.18-0.38) | 0.24(0.18-0.33) | 0.0811 |
| CV terminal P wave area | -2.18(-3.52- -1.65) | -2.2(-3.85 - -1.5) | -2.18(-3.33 - -1.71) | 0.8797 |

AF: atrial fibrillation; COPD: chronic obstructive pulmonary disease; IHD: ischemic heart disease; PVD: peripheral vascular disease; TIA: transient ischemic attack; ACEI: angiotensin-converting enzyme inhibitor; ARB: angiotensin II receptor blocker; SGLT2: sodium-glucose co-transporter 2; DPP-4: dipeptidyl peptidase-4 inhibitors; SD: standard deviation; CV: coefficient of variation (mean / standard deviation); P’ wave: component of the P wave below the isoelectric line

* for p ≤ 0.05, ** for p ≤ 0.01, *** for p ≤ 0.001.

# indicates that the comparisons were made between patients meeting primary new onset AF vs. those that did not.

**Supplementary Table 3. Baseline clinical characteristics of study population stratified by maximum P wave duration**

* for p ≤ 0.05, ** for p ≤ 0.01, *** for p ≤ 0.001

| **Characteristics** | **Overall**  **(N=2699)**  **Median (IQR) or Count (%)** | **<90 ms**  **(N=149)**  **Median (IQR) or Count (%)** | **91-100 ms (N=272)**  **Median (IQR) or Count (%)** | **101-120 ms (N=1041)**  **Median (IQR) or Count (%)** | **P value** | **101-120 ms (N=1041)**  **Median (IQR) or Count (%)** | **131-140 ms (N=321)**  **Median (IQR) or Count (%)** | **>141 ms**  **(N=551) Median (IQR) or Count (%)** | **P value** |
| --- | --- | --- | --- | --- | --- | --- | --- | --- | --- |
| ***Outcomes*** |  |  |  |  |  |  |  |  |  |
| New onset AF | 1140(42.23%) | 66(44.29%) | 121(44.48%) | 390(37.46%) | 0.2751 | 152(42.81%) | 147(45.79%) | 257(46.64%) | 0.779 |
| Time to new onset AF | 1567.0(602.5-2985.5);n=2699 | 1248.0(390.0-2389.0);n=149 | 1831.5(744.0-3582.0);n=272 | 1827.0(698.0-3330.0);n=1041 | 0.0096** | 1620.0(682.0-2961.5);n=355 | 1436.0(591.0-2560.0);n=321 | 1167.0(438.0-2469.5);n=551 | 0.0008*** |
| Stroke | 337(12.48%) | 18(12.08%) | 42(15.44%) | 122(11.71%) | 0.3486 | 56(15.77%) | 39(12.14%) | 60(10.88%) | 0.1616 |
| Time to stroke | 1591.0(590.0-3174.5);n=2699 | 1199.0(356.0-2577.0);n=149 | 1814.5(744.0-3582.0);n=272 | 1889.0(690.0-3496.0);n=1041 | 0.0035** | 1582.0(603.5-3142.5);n=355 | 1517.0(635.0-2908.0);n=321 | 1225.0(432.5-2543.5);n=551 | 0.0016** |
| Cardiovascular mortality | 556(20.60%) | 40(26.84%) | 57(20.95%) | 211(20.26%) | 0.3456 | 78(21.97%) | 57(17.75%) | 110(19.96%) | 0.5346 |
| Time to cardiovascular mortality | 1754.0(698.5-3297.0);n=2699 | 1359.0(463.0-2916.0);n=149 | 2088.0(845.0-3719.0);n=272 | 2069.0(841.0-3543.0);n=1041 | 0.0035** | 1785.0(754.5-3246.5);n=355 | 1610.0(636.0-2977.0);n=321 | 1397.0(510.0-2675.5);n=551 | 0.0007*** |
| All-cause mortality | 1958(72.54%) | 119(79.86%) | 185(68.01%) | 724(69.54%) | 0.5359 | 262(73.80%) | 244(76.01%) | 419(76.04%) | 0.9534 |
| Time to all-cause mortality | 1754.0(698.5-3297.0);n=2699 | 1359.0(463.0-2916.0);n=149 | 2088.0(845.0-3719.0);n=272 | 2069.0(841.0-3543.0);n=1041 | 0.0035** | 1785.0(754.5-3246.5);n=355 | 1610.0(636.0-2977.0);n=321 | 1397.0(510.0-2675.5);n=551 | 0.0007*** |
| ***Demographics*** |  |  |  |  |  |  |  |  |  |
| Male gender | 1298(48.09%) | 77(51.67%) | 130(47.79%) | 541(51.96%) | 0.7787 | 165(46.47%) | 137(42.67%) | 247(44.82%) | 0.8278 |
| Baseline age, years | 77.36(66.92-84.29);n=2699 | 78.01(65.07-84.39);n=149 | 74.66(63.06-82.32);n=272 | 75.58(64.8-82.92);n=1041 | 0.1912 | 76.69(65.7-83.41);n=355 | 80.06(72.91-86.68);n=321 | 80.18(71.57-86.9);n=551 | <0.0001*** |
| <50 | 124(4.59%) | 9(6.04%) | 18(6.61%) | 62(5.95%) | 0.9295 | 16(4.50%) | 5(1.55%) | 13(2.35%) | 0.0578 |
| [50-60] | 256(9.48%) | 13(8.72%) | 33(12.13%) | 115(11.04%) | 0.6286 | 37(10.42%) | 21(6.54%) | 35(6.35%) | 0.0869 |
| [60-70] | 447(16.56%) | 32(21.47%) | 56(20.58%) | 186(17.86%) | 0.5326 | 68(19.15%) | 35(10.90%) | 69(12.52%) | 0.0151* |
| [70-80] | 769(28.49%) | 31(20.80%) | 73(26.83%) | 312(29.97%) | 0.1845 | 96(27.04%) | 98(30.52%) | 155(28.13%) | 0.7468 |
| >80 | 1103(40.86%) | 64(42.95%) | 92(33.82%) | 366(35.15%) | 0.4067 | 138(38.87%) | 162(50.46%) | 279(50.63%) | 0.0761 |
| Charlson score | 5.0(4.0-6.0);n=2699 | 5.0(3.0-7.0);n=149 | 5.0(3.0-6.0);n=272 | 5.0(4.0-6.0);n=1041 | 0.0796 | 5.0(4.0-7.0);n=355 | 5.0(4.0-7.0);n=321 | 5.0(4.0-7.0);n=551 | 0.0047** |
| CHA2DS2-VASc score | 4.0(2.0-5.0);n=2699 | 4.0(2.0-5.0);n=149 | 3.0(2.0-5.0);n=272 | 3.0(2.0-5.0);n=1041 | 0.4433 | 3.0(2.0-5.0);n=355 | 4.0(3.0-5.0);n=321 | 4.0(3.0-5.0);n=551 | <0.0001*** |
| ***Past comorbidities*** |  |  |  |  |  |  |  |  |  |
| Diabetes without chronic complication | 786(29.12%) | 45(30.20%) | 77(28.30%) | 320(30.73%) | 0.8491 | 100(28.16%) | 92(28.66%) | 152(27.58%) | 0.9668 |
| Diabetes with chronic complication | 255(9.44%) | 15(10.06%) | 23(8.45%) | 102(9.79%) | 0.814 | 35(9.85%) | 29(9.03%) | 49(8.89%) | 0.899 |
| Renal diseases | 344(12.74%) | 18(12.08%) | 32(11.76%) | 129(12.39%) | 0.968 | 46(12.95%) | 48(14.95%) | 68(12.34%) | 0.6256 |
| Systemic embolism | 7(0.25%) | 0(0.00%) | 0(0.00%) | 3(0.28%) | 0.5455 | 0(0.00%) | 0(0.00%) | 4(0.72%) | 0.0868 |
| Hypertension | 1247(46.20%) | 64(42.95%) | 111(40.80%) | 467(44.86%) | 0.7435 | 164(46.19%) | 162(50.46%) | 277(50.27%) | 0.7408 |
| Prior heart failure | 2039(75.54%) | 112(75.16%) | 191(70.22%) | 722(69.35%) | 0.8351 | 278(78.30%) | 257(80.06%) | 473(85.84%) | 0.6236 |
| Myocardial infarction | 352(13.04%) | 17(11.40%) | 37(13.60%) | 151(14.50%) | 0.6595 | 38(10.70%) | 51(15.88%) | 57(10.34%) | 0.078 |
| Chronic renal failure | 72(2.66%) | 3(2.01%) | 5(1.83%) | 30(2.88%) | 0.5773 | 7(1.97%) | 10(3.11%) | 15(2.72%) | 0.6459 |
| Liver diseases | 15(0.55%) | 0(0.00%) | 0(0.00%) | 4(0.38%) | 0.4458 | 1(0.28%) | 4(1.24%) | 6(1.08%) | 0.3413 |
| Ventricular tachycardia/fibrillation | 132(4.89%) | 8(5.36%) | 9(3.30%) | 54(5.18%) | 0.4497 | 12(3.38%) | 18(5.60%) | 31(5.62%) | 0.2944 |
| Dementia and Alzheimer | 14(0.51%) | 0(0.00%) | 0(0.00%) | 5(0.48%) | 0.3643 | 4(1.12%) | 0(0.00%) | 5(0.90%) | 0.1901 |
| COPD | 386(14.30%) | 20(13.42%) | 35(12.86%) | 146(14.02%) | 0.9062 | 47(13.23%) | 52(16.19%) | 85(15.42%) | 0.6155 |
| IHD | 1008(37.34%) | 51(34.22%) | 103(37.86%) | 411(39.48%) | 0.6955 | 136(38.30%) | 123(38.31%) | 181(32.84%) | 0.3928 |
| PVD | 66(2.44%) | 2(1.34%) | 5(1.83%) | 26(2.49%) | 0.6026 | 9(2.53%) | 11(3.42%) | 13(2.35%) | 0.6453 |
| Stroke/TIA | 362(13.41%) | 22(14.76%) | 31(11.39%) | 126(12.10%) | 0.6597 | 50(14.08%) | 45(14.01%) | 88(15.97%) | 0.7223 |
| Gastrointestinal bleeding | 335(12.41%) | 14(9.39%) | 29(10.66%) | 121(11.62%) | 0.738 | 42(11.83%) | 49(15.26%) | 79(14.33%) | 0.4919 |
| Cancer | 162(6.00%) | 11(7.38%) | 10(3.67%) | 63(6.05%) | 0.2513 | 19(5.35%) | 22(6.85%) | 37(6.71%) | 0.6853 |
| Obesity | 44(1.63%) | 2(1.34%) | 4(1.47%) | 19(1.82%) | 0.867 | 5(1.40%) | 5(1.55%) | 9(1.63%) | 0.9657 |
| ***Medications*** |  |  |  |  |  |  |  |  |  |
| ACEI | 1250(46.31%) | 76(51.00%) | 119(43.75%) | 475(45.62%) | 0.6832 | 171(48.16%) | 146(45.48%) | 260(47.18%) | 0.9138 |
| ARB | 254(9.41%) | 17(11.40%) | 26(9.55%) | 97(9.31%) | 0.765 | 39(10.98%) | 21(6.54%) | 48(8.71%) | 0.175 |
| Calcium channel blockers | 1223(45.31%) | 63(42.28%) | 123(45.22%) | 473(45.43%) | 0.9035 | 156(43.94%) | 155(48.28%) | 248(45.00%) | 0.7711 |
| Beta blockers | 1325(49.09%) | 73(48.99%) | 124(45.58%) | 500(48.03%) | 0.8933 | 192(54.08%) | 155(48.28%) | 275(49.90%) | 0.6654 |
| Diuretics for hypertension | 317(11.74%) | 9(6.04%) | 31(11.39%) | 128(12.29%) | 0.1251 | 37(10.42%) | 46(14.33%) | 66(11.97%) | 0.3866 |
| Diuretics for heart failure | 1084(40.16%) | 72(48.32%) | 113(41.54%) | 370(35.54%) | 0.0966 | 146(41.12%) | 130(40.49%) | 249(45.19%) | 0.6206 |
| Nitrates | 747(27.67%) | 38(25.50%) | 71(26.10%) | 276(26.51%) | 0.9775 | 106(29.85%) | 104(32.39%) | 150(27.22%) | 0.482 |
| Antihypertensive drugs | 509(18.85%) | 29(19.46%) | 51(18.75%) | 182(17.48%) | 0.8402 | 72(20.28%) | 59(18.38%) | 113(20.50%) | 0.81 |
| Statins and fibrates | 825(30.56%) | 36(24.16%) | 96(35.29%) | 348(33.42%) | 0.2015 | 110(30.98%) | 92(28.66%) | 141(25.58%) | 0.4039 |
| Lipid-lowering drugs | 645(23.89%) | 28(18.79%) | 75(27.57%) | 282(27.08%) | 0.2219 | 81(22.81%) | 71(22.11%) | 106(19.23%) | 0.5239 |
| Anticoagulants | 500(18.52%) | 29(19.46%) | 43(15.80%) | 173(16.61%) | 0.7113 | 55(15.49%) | 72(22.42%) | 125(22.68%) | 0.0715 |
| Antiplatelets | 1108(41.05%) | 56(37.58%) | 111(40.80%) | 420(40.34%) | 0.9023 | 153(43.09%) | 139(43.30%) | 227(41.19%) | 0.9036 |
| Sodium-glucose cotransporter 2 inhibitors | 44(1.63%) | 5(3.35%) | 4(1.47%) | 24(2.30%) | 0.4686 | 1(0.28%) | 3(0.93%) | 6(1.08%) | 0.408 |
| Dipeptidyl peptidase-4 inhibitors | 206(7.63%) | 10(6.71%) | 25(9.19%) | 85(8.16%) | 0.713 | 24(6.76%) | 16(4.98%) | 45(8.16%) | 0.2454 |
| Alpha-glucosidase inhibitors | 69(2.55%) | 4(2.68%) | 9(3.30%) | 25(2.40%) | 0.7164 | 10(2.81%) | 9(2.80%) | 11(1.99%) | 0.6689 |
| ***Complete blood counts*** |  |  |  |  |  |  |  |  |  |
| Haemoglobin, g/dL | 11.8(10.4-13.3);n=1360 | 11.6(9.8-13.2);n=72 | 12.0(10.15-13.4);n=159 | 12.1(10.7-13.4);n=560 | 0.4566 | 11.6(10.1-13.5);n=170 | 11.65(10.3-13.0);n=156 | 11.55(9.95-12.9);n=238 | 0.6202 |
| Mean corpuscular volume, fL | 92.4(87.9-96.0);n=1360 | 92.9(88.35-97.2);n=72 | 92.2(88.1-95.35);n=159 | 92.2(87.5-96.0);n=560 | 0.485 | 91.85(87.75-96.6);n=170 | 93.6(88.3-96.4);n=156 | 92.3(87.95-95.5);n=238 | 0.4249 |
| Basophil, x10^9/L | 0.0(0.0-0.03);n=264 | 0.0(0.0-0.04);n=14 | 0.01(0.0-0.05);n=27 | 0.0(0.0-0.03);n=127 | 0.5419 | 0.0(0.0-0.02);n=42 | 0.01(0.0-0.02);n=22 | 0.01(0.0-0.02);n=32 | 0.4978 |
| Eosinophil, x10^9/L | 0.1(0.1-0.2);n=1178 | 0.1(0.08-0.26);n=63 | 0.1(0.07-0.3);n=137 | 0.1(0.1-0.3);n=475 | 0.39 | 0.1(0.1-0.2);n=151 | 0.1(0.0-0.2);n=135 | 0.1(0.03-0.2);n=214 | 0.3688 |
| Lymphocyte, x10^9/L | 1.2(0.8-1.68);n=1182 | 1.1(0.7-1.7);n=63 | 1.3(0.8-1.7);n=137 | 1.3(0.87-1.65);n=476 | 0.624 | 1.2(0.8-1.8);n=151 | 1.1(0.75-1.59);n=135 | 1.1(0.7-1.6);n=217 | 0.2022 |
| Blast, x10^9/L | 0.0(0.0-0.1);n=1012 | 0.0(0.0-0.05);n=51 | 0.0(0.0-0.1);n=121 | 0.0(0.0-0.1);n=394 | 0.9006 | 0.0(0.0-0.1);n=127 | 0.0(0.0-0.0);n=123 | 0.0(0.0-0.1);n=193 | 0.5675 |
| Metamyelocyte, x10^9/L | 0.12(0.06-0.22);n=32 | 1.2(1.2-1.2);n=1 | 0.09(0.08-0.21);n=5 | 0.09(0.06-0.14);n=11 | 0.2118 | 0.12(0.1-0.29);n=5 | 0.2(0.12-0.37);n=4 | 0.2(0.1-0.28);n=6 | 0.944 |
| Monocyte, x10^9/L | 0.5(0.4-0.7);n=1182 | 0.6(0.4-0.79);n=63 | 0.5(0.4-0.7);n=137 | 0.5(0.4-0.7);n=476 | 0.7592 | 0.5(0.4-0.7);n=151 | 0.6(0.4-0.7);n=135 | 0.5(0.4-0.7);n=217 | 0.5558 |
| Neutrophil, x10^9/L | 5.0(3.8-6.9);n=1182 | 5.3(4.0-8.4);n=63 | 5.0(3.9-6.4);n=137 | 4.97(3.72-7.0);n=476 | 0.3993 | 4.8(3.7-6.5);n=151 | 5.3(3.92-6.8);n=135 | 5.1(3.7-6.9);n=217 | 0.3651 |
| White cell count, x10^9/L | 7.2(5.8-9.0);n=1360 | 7.7(6.1-9.85);n=72 | 7.2(5.75-8.55);n=159 | 7.08(5.8-9.0);n=560 | 0.364 | 7.1(5.9-8.65);n=170 | 7.3(5.8-8.95);n=156 | 7.2(5.55-8.95);n=238 | 0.8192 |
| Mean cell haemoglobin, pg | 31.4(29.6-32.9);n=1360 | 31.6(29.55-33.4);n=72 | 31.5(30.05-32.7);n=159 | 31.4(29.5-32.8);n=560 | 0.4062 | 31.5(29.6-32.95);n=170 | 31.6(29.95-32.9);n=156 | 31.4(29.85-32.85);n=238 | 0.8158 |
| Myelocyte, x10^9/L | 0.08(0.04-0.2);n=23 | 0.32(0.32-0.32);n=2 | 0.14(0.14-0.14);n=2 | 0.07(0.03-0.12);n=9 | 0.3311 | 0.46(0.46-0.46);n=2 | 0.05(0.03-0.16);n=4 | 0.13(0.05-0.25);n=4 | 0.5737 |
| Platelet, x10^9/L | 196.0(153.0-239.0);n=1359 | 201.5(170.5-240.0);n=72 | 199.5(159.0-251.0);n=158 | 196.0(153.0-242.5);n=560 | 0.5922 | 191.5(158.0-232.0);n=170 | 198.0(149.0-238.5);n=156 | 186.0(147.0-232.0);n=238 | 0.699 |
| Reticulocyte, x10^9/L | 42.25(29.26-59.03);n=22 | 20.3(20.3-20.3);n=1 | 94.1(94.1-94.1);n=2 | 50.8(27.95-66.68);n=8 | 0.2084 | 51.79(48.39-58.74);n=3 | 55.7(55.7-55.7);n=2 | 30.95(29.26-32.4);n=6 | 0.1587 |
| Red blood count, x10^12/L | 3.89(3.36-4.38);n=1360 | 3.79(3.28-4.41);n=72 | 3.95(3.36-4.39);n=159 | 3.95(3.48-4.4);n=560 | 0.4271 | 3.84(3.36-4.44);n=170 | 3.85(3.32-4.38);n=156 | 3.78(3.27-4.3);n=238 | 0.5507 |
| Hematocrit, L/L | 0.38(0.32-0.4);n=42 | 0.44(0.36-0.44);n=3 | 0.4(0.39-0.41);n=3 | 0.38(0.34-0.4);n=21 | 0.328 | 0.35(0.32-0.38);n=6 | 0.32(0.29-0.4);n=5 | 0.33(0.22-0.34);n=4 | 0.6873 |
| ***Liver and renal functions*** |  |  |  |  |  |  |  |  |  |
| Potassium, mmol/L | 4.2(3.8-4.6);n=1616 | 4.1(3.98-4.45);n=83 | 4.2(3.8-4.5);n=186 | 4.2(3.9-4.6);n=669 | 0.263 | 4.2(3.8-4.5);n=208 | 4.2(3.8-4.5);n=192 | 4.19(3.7-4.56);n=272 | 0.5879 |
| Urate, mmol/L | 0.46(0.38-0.56);n=466 | 0.5(0.4-0.56);n=19 | 0.42(0.34-0.5);n=55 | 0.46(0.38-0.56);n=204 | 0.059 | 0.5(0.42-0.6);n=61 | 0.49(0.4-0.56);n=50 | 0.45(0.37-0.57);n=75 | 0.3092 |
| Albumin, g/L | 39.6(35.8-42.7);n=1504 | 39.2(33.8-42.8);n=79 | 40.6(36.9-43.2);n=173 | 40.0(36.3-42.6);n=626 | 0.32 | 40.0(35.7-42.73);n=192 | 39.0(35.1-42.4);n=177 | 38.55(34.8-42.35);n=252 | 0.1023 |
| Sodium, mmol/L | 140.5(138.2-142.4);n=1617 | 140.2(137.3-142.0);n=83 | 140.95(138.45-142.75);n=186 | 140.35(138.25-142.2);n=670 | 0.0445* | 140.35(138.3-142.2);n=208 | 141.0(138.4-143.0);n=192 | 140.2(137.9-142.25);n=272 | 0.0221* |
| Urea, mmol/L | 7.85(5.9-12.1);n=1617 | 8.1(5.98-13.7);n=83 | 7.28(5.5-11.46);n=186 | 7.8(5.86-11.8);n=670 | 0.413 | 7.7(5.8-13.0);n=208 | 8.56(6.19-12.3);n=192 | 8.1(5.82-11.95);n=272 | 0.5276 |
| Protein, g/L | 72.6(68.0-76.4);n=1488 | 72.0(68.1-75.0);n=78 | 73.1(69.0-76.3);n=173 | 72.8(68.35-76.3);n=619 | 0.4409 | 73.05(68.05-77.35);n=190 | 71.7(67.1-76.1);n=173 | 72.25(67.85-76.0);n=250 | 0.107 |
| Creatinine, umol/L | 110.0(85.0-153.0);n=1617 | 118.0(93.5-147.0);n=83 | 100.5(82.0-148.0);n=186 | 112.0(85.5-153.0);n=670 | 0.0441* | 108.0(85.0-167.0);n=208 | 110.5(86.0-153.0);n=192 | 108.0(83.0-151.5);n=272 | 0.4165 |
| Alkaline phosphatase, U/L | 77.0(62.0-99.0);n=1504 | 79.0(65.0-107.5);n=79 | 74.0(61.0-94.0);n=173 | 77.0(62.0-98.0);n=626 | 0.174 | 77.0(63.0-98.5);n=192 | 81.0(67.0-100.0);n=177 | 78.0(61.0-106.0);n=252 | 0.5089 |
| Aspartate transaminase, U/L | 26.5(20.0-38.0);n=78 | 25.5(20.5-33.0);n=6 | 34.0(24.0-47.5);n=7 | 24.5(18.5-41.5);n=36 | 0.7146 | 31.0(15.0-41.0);n=11 | 26.0(23.5-33.0);n=7 | 27.0(24.0-34.0);n=11 | 0.8989 |
| Alanine transaminase, U/L | 19.0(14.0-27.0);n=1425 | 18.0(13.5-23.5);n=76 | 20.0(16.0-28.0);n=167 | 19.0(13.0-27.0);n=580 | 0.0350* | 18.0(14.0-27.0);n=184 | 19.0(15.0-28.0);n=169 | 20.0(15.0-27.0);n=244 | 0.5 |
| Bilirubin, umol/L | 10.6(7.6-15.0);n=1499 | 11.0(8.0-14.5);n=79 | 10.0(8.0-15.2);n=173 | 10.3(7.3-14.5);n=624 | 0.7071 | 10.0(8.0-15.05);n=191 | 10.0(7.0-14.9);n=175 | 11.55(8.0-16.0);n=252 | 0.0561 |
| ***Lipid and glucose tests*** |  |  |  |  |  |  |  |  |  |
| Triglyceride, mmol/L | 1.19(0.88-1.74);n=1023 | 1.17(0.86-1.56);n=42 | 1.15(0.84-1.72);n=124 | 1.19(0.88-1.78);n=466 | 0.8493 | 1.25(0.96-1.98);n=138 | 1.08(0.82-1.63);n=111 | 1.14(0.86-1.51);n=138 | 0.0092** |
| Low-density lipoprotein, mmol/L | 2.28(1.8-2.9);n=992 | 2.18(1.82-2.75);n=40 | 2.32(1.72-2.89);n=116 | 2.26(1.78-2.83);n=456 | 0.9376 | 2.45(1.93-3.1);n=136 | 2.28(1.81-2.91);n=104 | 2.24(1.72-2.79);n=136 | 0.0669 |
| High-density lipoprotein, mmol/L | 1.25(1.0-1.54);n=1000 | 1.22(0.97-1.41);n=41 | 1.25(1.02-1.62);n=116 | 1.23(1.0-1.54);n=461 | 0.2392 | 1.27(1.0-1.5);n=137 | 1.35(1.03-1.61);n=105 | 1.19(1.0-1.49);n=136 | 0.232 |
| Cholesterol, mmol/L | 4.3(3.6-5.0);n=1023 | 4.02(3.66-4.64);n=42 | 4.23(3.43-5.04);n=124 | 4.3(3.56-4.98);n=466 | 0.8217 | 4.5(3.82-5.38);n=138 | 4.3(3.66-4.99);n=111 | 4.03(3.5-4.88);n=138 | 0.0032** |
| Glucose, mmol/L | 6.3(5.4-8.22);n=1306 | 6.69(5.74-8.56);n=70 | 6.26(5.5-8.1);n=152 | 6.4(5.4-8.3);n=556 | 0.4285 | 5.93(5.1-7.34);n=160 | 6.2(5.41-8.08);n=152 | 6.43(5.5-8.32);n=211 | 0.0646 |
| ***Cardiac, clotting, inflammatory,***  ***and acid-base tests*** |  |  |  |  |  |  |  |  |  |
| D-dimer, ng/mL | 1315.21(747.53-2692.78);n=309 | 1711.75(1211.2-3723.3);n=23 | 1203.5(673.98-2867.83);n=31 | 1273.18(773.67-2570.02);n=118 | 0.232 | 1133.38(647.76-3278.27);n=40 | 1866.95(782.56-2995.6);n=35 | 1342.21(830.01-2347.27);n=61 | 0.7484 |
| High sensitive troponin-I, ng/L | 0.03(0.02-0.07);n=1872 | 0.03(0.02-0.07);n=93 | 0.03(0.02-0.06);n=188 | 0.03(0.02-0.07);n=704 | 0.2979 | 0.03(0.02-0.07);n=253 | 0.03(0.02-0.06);n=239 | 0.03(0.02-0.07);n=392 | 0.8686 |
| Lactate dehydrogenase, U/L | 237.0(194.0-303.0);n=2334 | 244.0(195.0-311.0);n=129 | 235.5(191.5-301.5);n=230 | 234.0(192.0-309.5);n=918 | 0.5414 | 245.0(198.5-313.5);n=311 | 232.0(193.0-293.0);n=279 | 239.5(202.0-292.0);n=458 | 0.0471* |
| APTT, second | 33.4(29.9-39.8);n=1015 | 33.7(30.6-41.4);n=53 | 33.6(30.05-39.85);n=112 | 32.9(29.5-38.5);n=395 | 0.24 | 33.0(30.25-38.4);n=131 | 34.0(30.0-39.7);n=125 | 35.7(30.7-42.1);n=197 | 0.0312* |
| Prothrombin time/INR, second | 12.4(10.9-19.95);n=971 | 12.9(11.15-21.1);n=50 | 12.3(10.9-17.55);n=107 | 12.1(10.5-19.0);n=371 | 0.2663 | 12.2(10.8-19.45);n=127 | 12.1(10.95-19.9);n=123 | 13.15(11.2-22.8);n=190 | 0.0421* |
| ***ECG measurements*** |  |  |  |  |  |  |  |  |  |
| Trans QRS initangle | 43.5(21.0-291.5);n=896 | 65.0(26.0-321.5);n=47 | 60.0(23.0-333.0);n=49 | 44.0(19.0-298.5);n=235 | 0.2116 | 40.0(19.0-254.0);n=109 | 41.0(24.0-236.0);n=145 | 43.0(21.0-273.0);n=309 | 0.9427 |
| Trans QRS initmag | 0.4(0.23-0.64);n=896 | 0.43(0.23-0.72);n=47 | 0.43(0.27-0.61);n=49 | 0.4(0.23-0.61);n=235 | 0.9548 | 0.4(0.24-0.62);n=109 | 0.37(0.21-0.56);n=145 | 0.4(0.22-0.67);n=309 | 0.2665 |
| Trans QRS max angle | 316.5(29.0-345.0);n=896 | 306.0(204.0-346.0);n=47 | 322.0(277.0-345.0);n=49 | 315.0(31.0-344.0);n=235 | 0.283 | 322.0(114.0-345.0);n=109 | 323.0(24.0-347.0);n=145 | 313.0(18.0-345.0);n=309 | 0.5701 |
| Trans QRS max mag | 1.28(0.98-1.74);n=896 | 1.32(1.0-1.54);n=47 | 1.26(0.99-1.67);n=49 | 1.22(0.95-1.71);n=235 | 0.8802 | 1.33(1.0-1.71);n=109 | 1.22(0.94-1.58);n=145 | 1.37(1.01-1.83);n=309 | 0.0333* |
| Trans QRS term angle | 235.0(203.5-267.5);n=896 | 229.0(204.0-254.0);n=47 | 238.0(209.0-270.0);n=49 | 233.0(199.5-258.5);n=235 | 0.677 | 239.0(207.0-261.0);n=109 | 234.0(207.0-270.0);n=145 | 238.0(205.0-273.0);n=309 | 0.7925 |
| Trans QRS term mag | 0.35(0.24-0.49);n=896 | 0.41(0.3-0.52);n=47 | 0.33(0.26-0.54);n=49 | 0.34(0.22-0.48);n=235 | 0.1854 | 0.33(0.25-0.47);n=109 | 0.33(0.22-0.46);n=145 | 0.36(0.24-0.5);n=309 | 0.2182 |
| Trans QRS cwrot | -100.0(-100.0--7.5);n=896 | -100.0(-100.0-0.0);n=47 | -100.0(-100.0--43.0);n=49 | -100.0(-100.0-0.0);n=235 | 0.4278 | -100.0(-100.0--100.0);n=109 | -100.0(-100.0--75.0);n=145 | -100.0(-100.0-0.0);n=309 | 0.0173* |
| P wave front axis | 55.0(34.0-72.0);n=2163 | 54.0(31.0-77.0);n=97 | 59.0(30.5-73.0);n=208 | 56.0(40.0-71.0);n=884 | 0.8518 | 56.0(35.0-70.0);n=301 | 56.0(37.0-72.0);n=249 | 49.0(23.0-74.0);n=417 | 0.1701 |
| P wave horizon axis | 21.0(1.0-47.0);n=2263 | 28.0(0.0-63.0);n=103 | 25.0(6.5-53.0);n=227 | 21.0(4.0-42.0);n=918 | 0.132 | 20.0(2.0-39.0);n=317 | 18.0(-4.0-41.0);n=263 | 21.0(-6.0-56.0);n=426 | 0.6153 |
| I 40 front axis | 35.0(10.0-58.0);n=2601 | 41.5(14.0-63.5);n=140 | 36.0(9.5-60.0);n=262 | 34.0(7.5-56.0);n=1007 | 0.0899 | 33.5(11.0-56.5);n=346 | 34.0(8.0-59.0);n=305 | 35.0(11.0-59.0);n=531 | 0.8119 |
| I 40 horizon axis | 21.0(1.0-40.0);n=2494 | 21.0(-7.0-39.5);n=131 | 19.0(-5.0-37.5);n=247 | 22.0(3.0-40.0);n=970 | 0.2914 | 22.0(0.0-41.0);n=330 | 22.0(2.0-40.0);n=295 | 19.0(2.0-38.0);n=511 | 0.7434 |
| QRS front axis | 38.0(-5.0-71.0);n=2665 | 41.5(-23.0-79.5);n=146 | 44.0(2.0-73.5);n=268 | 35.0(-3.0-70.0);n=1028 | 0.2891 | 38.0(-10.0-64.0);n=353 | 35.0(-4.0-69.0);n=316 | 37.0(-8.0-74.0);n=545 | 0.8807 |
| QRS horizon axis | -12.0(-38.0-14.0);n=2525 | -16.0(-46.0-36.0);n=131 | -12.0(-37.0-11.0);n=254 | -12.0(-36.0-14.0);n=977 | 0.9753 | -13.0(-41.0-7.5);n=336 | -12.0(-39.0-13.0);n=301 | -11.0(-37.0-16.0);n=516 | 0.3437 |
| T wave 40ms front axis | 57.0(-20.0-107.0);n=2605 | 65.0(-41.5-126.5);n=143 | 58.0(-3.5-103.0);n=262 | 59.0(-17.5-104.5);n=1003 | 0.8478 | 47.5(-29.0-90.5);n=348 | 57.0(-15.0-121.0);n=309 | 56.0(-25.0-116.0);n=531 | 0.0998 |
| T wave 40ms horizon axis | -17.5(-55.0-226.0);n=2534 | 134.0(-40.0-238.5);n=134 | -21.0(-57.0-191.0);n=253 | -20.0(-58.0-224.0);n=986 | 0.0084** | -23.5(-58.0-226.0);n=330 | -11.0(-53.5-225.5);n=300 | -12.5(-50.0-226.0);n=522 | 0.2264 |
| ST segment front axis | 106.0(42.0-181.5);n=2378 | 118.0(45.5-173.0);n=131 | 102.0(35.0-187.0);n=225 | 102.5(43.5-167.0);n=912 | 0.8699 | 105.0(41.0-190.0);n=309 | 105.0(31.5-183.5);n=288 | 116.0(44.5-197.5);n=504 | 0.545 |
| ST segment horizon axis | 106.0(68.0-153.0);n=2346 | 110.0(74.5-156.0);n=120 | 98.0(63.0-156.0);n=229 | 104.0(72.0-146.0);n=923 | 0.2747 | 106.0(71.0-148.0);n=313 | 110.5(65.5-158.5);n=280 | 109.0(63.5-160.0);n=471 | 0.7093 |
| T wave front axis | 60.0(20.0-106.0);n=2572 | 75.0(33.0-109.5);n=139 | 50.0(12.5-102.5);n=255 | 63.0(22.0-103.0);n=998 | 0.0678 | 55.0(24.0-109.5);n=340 | 57.0(18.0-101.0);n=305 | 60.0(19.0-111.0);n=525 | 0.5781 |
| T wave horizon axis | 63.0(33.0-114.0);n=2421 | 66.0(38.0-104.0);n=129 | 61.5(33.5-118.5);n=236 | 66.0(39.0-116.0);n=942 | 0.4626 | 68.0(34.0-117.0);n=327 | 63.0(27.0-105.5);n=292 | 57.0(29.0-114.0);n=485 | 0.1814 |
| Atrial rate | 75.0(61.0-96.0);n=2699 | 75.0(50.0-111.0);n=149 | 78.0(66.0-103.0);n=272 | 74.0(62.0-92.0);n=1041 | 0.0152* | 75.0(62.0-91.0);n=355 | 77.0(59.0-95.0);n=321 | 75.0(59.5-100.0);n=551 | 0.8342 |
| Mean ventricular rate | 77.0(66.0-90.0);n=2699 | 84.0(68.0-111.0);n=149 | 81.0(70.0-99.0);n=272 | 76.0(66.0-89.0);n=1041 | <0.0001*** | 77.0(66.0-87.0);n=355 | 78.0(67.0-88.0);n=321 | 75.0(65.0-87.0);n=551 | 0.4871 |
| Mean PT interval | 176.0(158.0-199.5);n=2699 | 143.0(127.0-166.0);n=149 | 162.0(148.5-176.0);n=272 | 172.0(157.0-188.0);n=1041 | <0.0001*** | 183.0(166.5-203.0);n=355 | 188.0(168.0-210.0);n=321 | 196.0(168.0-228.0);n=551 | <0.0001*** |
| Mean PR segment | 79.0(64.0-99.0);n=2699 | 72.0(58.0-94.0);n=149 | 79.0(68.0-92.0);n=272 | 77.0(64.0-94.0);n=1041 | 0.0241* | 78.0(63.0-99.0);n=355 | 82.0(64.0-104.0);n=321 | 83.0(62.0-112.0);n=551 | 0.0757 |
| Mean QRS duration | 91.0(82.5-108.0);n=2699 | 97.0(83.0-132.0);n=149 | 88.0(79.0-101.0);n=272 | 91.0(82.0-106.0);n=1041 | <0.0001*** | 90.0(83.0-104.5);n=355 | 91.0(83.0-108.0);n=321 | 94.0(84.0-113.0);n=551 | 0.1387 |
| Mean QT interval | 393.0(360.0-428.0);n=2699 | 388.0(336.0-428.0);n=149 | 374.5(340.5-411.0);n=272 | 389.0(358.0-423.0);n=1041 | <0.0001*** | 400.0(364.0-428.5);n=355 | 398.0(369.0-432.0);n=321 | 404.0(372.0-444.0);n=551 | 0.0269* |
| Mean QTc | 444.0(420.0-472.0);n=2699 | 450.0(423.0-480.0);n=149 | 436.0(414.0-463.0);n=272 | 439.0(415.0-468.0);n=1041 | 0.0074** | 446.0(423.5-472.0);n=355 | 450.0(429.0-476.0);n=321 | 454.0(430.0-478.0);n=551 | 0.0232* |
| QT interval dispersion | 0.0(0.0-72.0);n=2699 | 0.0(0.0-64.0);n=149 | 0.0(0.0-0.0);n=272 | 0.0(0.0-0.0);n=1041 | 0.0061** | 0.0(0.0-56.0);n=355 | 0.0(0.0-88.0);n=321 | 60.0(0.0-108.0);n=551 | <0.0001*** |
| Mean p wave amplitude | 0.04(0.01-0.06);n=2699 | 0.02(0.0-0.05);n=149 | 0.04(0.01-0.06);n=272 | 0.05(0.02-0.06);n=1041 | <0.0001*** | 0.05(0.02-0.06);n=355 | 0.03(0.01-0.06);n=321 | 0.03(0.0-0.05);n=551 | <0.0001*** |
| Max p wave amplitude | 0.12(0.09-0.16);n=2699 | 0.11(0.08-0.16);n=149 | 0.12(0.1-0.16);n=272 | 0.13(0.1-0.16);n=1041 | 0.0174* | 0.13(0.1-0.17);n=355 | 0.12(0.08-0.16);n=321 | 0.11(0.08-0.14);n=551 | <0.0001*** |
| Min p wave amplitude | -0.1(-0.12--0.07);n=2699 | -0.09(-0.13--0.07);n=149 | -0.1(-0.13--0.08);n=272 | -0.1(-0.12--0.08);n=1041 | 0.3681 | -0.1(-0.13--0.08);n=355 | -0.09(-0.12--0.07);n=321 | -0.09(-0.12--0.07);n=551 | 0.0075** |
| Max-min p wave amplitude | 0.22(0.17-0.28);n=2699 | 0.21(0.15-0.27);n=149 | 0.22(0.17-0.28);n=272 | 0.23(0.18-0.29);n=1041 | 0.0351* | 0.23(0.17-0.29);n=355 | 0.21(0.16-0.28);n=321 | 0.2(0.16-0.25);n=551 | <0.0001*** |
| SD p wave amplitude | 0.07(0.05-0.09);n=2652 | 0.07(0.05-0.09);n=138 | 0.07(0.05-0.09);n=262 | 0.07(0.05-0.09);n=1028 | 0.6575 | 0.07(0.05-0.09);n=351 | 0.07(0.05-0.09);n=318 | 0.06(0.05-0.08);n=545 | 0.0001*** |
| CV p wave amplitude | 1.29(0.9-2.1);n=2643 | 1.46(0.83-2.87);n=137 | 1.31(0.93-1.91);n=262 | 1.27(0.94-1.92);n=1026 | 0.2148 | 1.28(0.96-1.94);n=349 | 1.24(0.87-2.38);n=315 | 1.3(0.7-2.37);n=544 | 0.6312 |
| Mean p wave duration | 78.83(67.58-87.79);n=2699 | 52.33(36.33-62.0);n=149 | 68.58(59.58-73.79);n=272 | 78.08(69.33-84.17);n=1041 | <0.0001*** | 84.67(76.29-91.92);n=355 | 84.0(73.67-94.0);n=321 | 89.0(76.71-99.67);n=551 | <0.0001*** |
| Max p wave duration.1 | 120.0(108.0-136.0);n=2699 | 82.0(76.0-87.0);n=149 | 96.0(93.0-100.0);n=272 | 112.0(108.0-116.0);n=1041 | <0.0001*** | 125.0(124.0-128.0);n=355 | 136.0(132.0-140.0);n=321 | 160.0(150.0-176.0);n=551 | <0.0001*** |
| Min p wave duration | 32.0(0.0-43.0);n=2699 | 16.0(0.0-32.0);n=149 | 30.0(0.0-38.5);n=272 | 36.0(24.0-44.0);n=1041 | <0.0001*** | 36.0(24.0-45.5);n=355 | 32.0(0.0-44.0);n=321 | 30.0(0.0-40.0);n=551 | <0.0001*** |
| Max-min p wave duration | 89.0(72.0-114.0);n=2699 | 61.0(49.0-80.0);n=149 | 66.0(58.0-92.0);n=272 | 78.0(68.0-92.0);n=1041 | <0.0001*** | 90.0(80.0-100.5);n=355 | 104.0(92.0-132.0);n=321 | 136.0(116.0-157.0);n=551 | <0.0001*** |
| SD p wave duration | 28.04(22.72-36.14);n=2681 | 19.83(15.61-26.92);n=145 | 21.85(18.6-26.75);n=267 | 24.68(21.16-29.35);n=1037 | <0.0001*** | 28.17(24.67-32.98);n=354 | 32.65(27.41-39.41);n=321 | 40.66(35.0-49.37);n=547 | <0.0001*** |
| CV p wave duration | 0.35(0.28-0.48);n=2681 | 0.37(0.25-0.81);n=145 | 0.31(0.26-0.42);n=267 | 0.32(0.26-0.4);n=1037 | 0.0024** | 0.33(0.28-0.41);n=354 | 0.38(0.31-0.51);n=321 | 0.45(0.37-0.6);n=547 | <0.0001*** |
| Mean p wave area | 0.42(0.14-0.63);n=2699 | 0.19(0.01-0.4);n=149 | 0.41(0.12-0.58);n=272 | 0.48(0.22-0.68);n=1041 | <0.0001*** | 0.48(0.27-0.74);n=355 | 0.37(0.08-0.65);n=321 | 0.32(0.0-0.55);n=551 | <0.0001*** |
| Max p wave area | 1.5(1.0-2.05);n=2699 | 1.0(0.6-1.4);n=149 | 1.3(0.9-1.9);n=272 | 1.6(1.1-2.1);n=1041 | <0.0001*** | 1.7(1.2-2.4);n=355 | 1.5(1.0-2.2);n=321 | 1.5(1.0-2.1);n=551 | 0.0012** |
| Min p wave area | -1.1(-1.5--0.8);n=2699 | -0.7(-1.1--0.5);n=149 | -1.0(-1.3--0.6);n=272 | -1.1(-1.5--0.8);n=1041 | <0.0001*** | -1.2(-1.7--0.8);n=355 | -1.1(-1.6--0.8);n=321 | -1.2(-1.7--0.8);n=551 | 0.3872 |
| Max-min p wave area | 2.6(1.9-3.6);n=2699 | 1.7(1.3-2.4);n=149 | 2.25(1.7-3.15);n=272 | 2.7(2.0-3.5);n=1041 | <0.0001*** | 2.9(2.1-4.0);n=355 | 2.7(2.0-3.8);n=321 | 2.8(2.1-3.7);n=551 | 0.1449 |
| SD p wave area | 0.75(0.56-0.99);n=2681 | 0.5(0.38-0.72);n=145 | 0.63(0.5-0.89);n=267 | 0.76(0.57-0.97);n=1037 | <0.0001*** | 0.83(0.63-1.12);n=354 | 0.76(0.56-1.06);n=321 | 0.81(0.62-1.08);n=547 | 0.1717 |
| CV p wave area | 1.39(1.03-2.11);n=2669 | 1.38(0.81-2.54);n=142 | 1.33(1.05-1.82);n=264 | 1.36(1.06-1.94);n=1035 | 0.8192 | 1.39(1.07-2.02);n=354 | 1.4(0.98-2.34);n=321 | 1.47(0.83-2.43);n=543 | 0.9576 |
| Mean p' wave amplitude | -0.01(-0.01--0.0);n=2065 | -0.0(-0.01-0.0);n=84 | -0.01(-0.01--0.0);n=199 | -0.01(-0.01--0.0);n=830 | 0.1528 | -0.01(-0.01--0.0);n=290 | -0.01(-0.01--0.0);n=247 | -0.01(-0.01--0.0);n=409 | 0.0020** |
| Max p' wave amplitude | 0.0(0.0-0.05);n=2065 | 0.02(0.0-0.06);n=84 | 0.01(0.0-0.07);n=199 | 0.0(0.0-0.05);n=830 | 0.0028** | 0.0(0.0-0.05);n=290 | 0.0(0.0-0.04);n=247 | 0.0(0.0-0.04);n=409 | 0.3266 |
| Min p' wave amplitude | -0.07(-0.09--0.05);n=2065 | -0.06(-0.1--0.04);n=84 | -0.07(-0.1--0.05);n=199 | -0.07(-0.1--0.05);n=830 | 0.7635 | -0.07(-0.1--0.05);n=290 | -0.07(-0.09--0.04);n=247 | -0.05(-0.08--0.04);n=409 | <0.0001*** |
| Max-min p' wave amplitude | 0.08(0.06-0.12);n=2065 | 0.1(0.06-0.15);n=84 | 0.09(0.06-0.14);n=199 | 0.09(0.06-0.12);n=830 | 0.0707 | 0.1(0.06-0.13);n=290 | 0.08(0.06-0.11);n=247 | 0.07(0.05-0.1);n=409 | <0.0001*** |
| SD p' wave amplitude | 0.03(0.03-0.04);n=799 | 0.04(0.03-0.05);n=31 | 0.04(0.03-0.05);n=79 | 0.04(0.03-0.05);n=319 | 0.1961 | 0.03(0.03-0.04);n=127 | 0.03(0.02-0.04);n=101 | 0.03(0.02-0.04);n=140 | 0.0003*** |
| CV p' wave amplitude | -1.99(-3.79--1.54);n=795 | -1.55(-2.63-1.86);n=31 | -2.27(-3.93--1.61);n=79 | -2.04(-3.84--1.58);n=318 | 0.0323* | -2.05(-4.03--1.56);n=124 | -1.96(-3.69--1.55);n=101 | -1.96(-3.6--1.36);n=140 | 0.4038 |
| Mean p' wave duration | 9.5(5.58-15.67);n=2047 | 8.67(4.58-13.5);n=84 | 9.0(5.5-14.5);n=195 | 9.08(5.25-14.67);n=823 | 0.662 | 10.33(5.67-16.67);n=289 | 10.67(5.92-17.5);n=244 | 10.0(5.67-16.62);n=406 | 0.8018 |
| Max p' wave duration | 65.0(52.0-80.0);n=2047 | 60.0(48.0-77.0);n=84 | 60.0(48.0-76.0);n=195 | 61.0(49.0-76.0);n=823 | 0.5616 | 68.0(56.0-80.0);n=289 | 72.0(56.0-85.5);n=244 | 75.0(56.0-92.0);n=406 | 0.0040** |
| Max-min p' wave duration | 65.0(52.0-80.0);n=2047 | 60.0(48.0-77.0);n=84 | 60.0(48.0-76.0);n=195 | 61.0(49.0-76.0);n=823 | 0.5616 | 68.0(56.0-80.0);n=289 | 72.0(56.0-85.5);n=244 | 75.0(56.0-92.0);n=406 | 0.0040** |
| SD p' wave duration | 28.63(24.21-34.02);n=796 | 26.94(21.49-31.24);n=31 | 26.0(22.99-30.21);n=79 | 27.11(22.98-32.41);n=317 | 0.1169 | 28.55(24.38-33.68);n=127 | 30.96(27.31-36.35);n=101 | 32.2(26.85-37.15);n=139 | 0.0007*** |
| CV p' wave duration | 1.66(1.4-1.88);n=796 | 1.81(1.3-1.9);n=31 | 1.6(1.32-1.89);n=79 | 1.81(1.44-1.88);n=317 | 0.9078 | 1.68(1.5-1.88);n=127 | 1.63(1.49-1.87);n=101 | 1.61(1.34-1.86);n=139 | 0.404 |
| Mean p wave duration+ppdur | 88.17(75.21-98.96);n=2699 | 59.67(38.5-71.58);n=149 | 76.62(65.08-83.25);n=272 | 87.25(77.42-94.67);n=1041 | <0.0001*** | 96.0(85.67-103.38);n=355 | 95.08(81.83-105.33);n=321 | 97.67(82.83-111.0);n=551 | 0.0036** |
| Max p wave duration+ppdur | 128.0(112.0-151.5);n=2699 | 88.0(80.0-112.0);n=149 | 100.0(96.0-120.0);n=272 | 116.0(110.0-126.0);n=1041 | <0.0001*** | 128.0(124.0-138.0);n=355 | 140.0(136.0-144.0);n=321 | 164.0(152.0-184.0);n=551 | <0.0001*** |
| Min p wave duration+ppdur | 44.0(0.0-60.0);n=2699 | 16.0(0.0-40.0);n=149 | 40.0(0.0-54.0);n=272 | 48.0(28.0-60.0);n=1041 | <0.0001*** | 52.0(32.0-66.0);n=355 | 43.0(0.0-60.0);n=321 | 36.0(0.0-56.0);n=551 | <0.0001*** |
| Max-min p wave duration+ppdur | 90.0(65.0-120.0);n=2699 | 76.0(53.0-88.0);n=149 | 68.0(52.0-96.0);n=272 | 72.0(58.0-104.0);n=1041 | 0.0052** | 81.0(64.0-116.0);n=355 | 100.0(84.0-136.0);n=321 | 136.0(108.0-160.0);n=551 | <0.0001*** |
| SD p wave duration+ppdur | 26.91(19.43-37.11);n=2681 | 24.69(17.4-31.69);n=145 | 20.24(15.19-30.84);n=267 | 22.05(17.21-30.78);n=1037 | 0.0095** | 24.75(19.55-33.14);n=354 | 31.15(24.33-40.41);n=321 | 39.49(31.26-51.82);n=547 | <0.0001*** |
| CV p wave duration+ppdur | 0.3(0.21-0.46);n=2681 | 0.42(0.27-0.84);n=145 | 0.25(0.19-0.43);n=267 | 0.25(0.19-0.38);n=1037 | <0.0001*** | 0.26(0.19-0.36);n=354 | 0.33(0.23-0.49);n=321 | 0.4(0.3-0.57);n=547 | <0.0001*** |
| Mean terminal p wave area | -0.04(-0.08--0.01);n=2045 | -0.02(-0.08-0.02);n=84 | -0.03(-0.07--0.01);n=195 | -0.04(-0.08--0.02);n=822 | 0.0030** | -0.05(-0.1--0.01);n=289 | -0.04(-0.1--0.01);n=244 | -0.03(-0.08-0.0);n=405 | 0.0280* |
| Max terminal p wave area | 0.0(0.0-0.3);n=2045 | 0.15(0.0-0.5);n=84 | 0.1(0.0-0.3);n=195 | 0.0(0.0-0.2);n=822 | 0.0018** | 0.0(0.0-0.3);n=289 | 0.0(0.0-0.3);n=244 | 0.0(0.0-0.3);n=405 | 0.9774 |
| Min terminal p wave area | -0.5(-0.8--0.2);n=2045 | -0.3(-0.7--0.1);n=84 | -0.4(-0.6--0.2);n=195 | -0.5(-0.7--0.2);n=822 | 0.0086** | -0.5(-0.8--0.3);n=289 | -0.5(-0.8--0.3);n=244 | -0.4(-0.7--0.2);n=405 | 0.0009*** |
| Max-min terminal p wave area | 0.6(0.3-1.0);n=2045 | 0.65(0.3-1.0);n=84 | 0.5(0.3-0.9);n=195 | 0.6(0.3-0.9);n=822 | 0.5527 | 0.7(0.4-1.1);n=289 | 0.6(0.4-1.0);n=244 | 0.6(0.3-0.9);n=405 | 0.0024** |
| SD terminal p wave area | 0.25(0.18-0.35);n=793 | 0.26(0.18-0.33);n=31 | 0.23(0.16-0.33);n=79 | 0.24(0.18-0.35);n=315 | 0.6447 | 0.25(0.2-0.36);n=126 | 0.25(0.18-0.38);n=101 | 0.25(0.18-0.34);n=139 | 0.6046 |
| CV terminal p wave area | -2.18(-3.52--1.65);n=774 | -1.6(-2.22-2.38);n=29 | -2.6(-4.13--1.94);n=77 | -2.28(-3.57--1.77);n=308 | 0.0012** | -2.2(-3.39--1.7);n=122 | -2.2(-3.56--1.64);n=99 |  |  |

AF: atrial fibrillation; COPD: chronic obstructive pulmonary disease; IHD: ischemic heart disease; PVD: peripheral vascular disease; TIA: transient ischemic attack; ACEI: angiotensin-converting enzyme inhibitor; ARB: angiotensin II receptor blocker; SGLT2: sodium-glucose co-transporter 2; DPP-4: dipeptidyl peptidase-4 inhibitors; SD: standard deviation; CV: coefficient of variation (mean / standard deviation); P’ wave: component of the P wave below the isoelectric line

* for p ≤ 0.05, ** for p ≤ 0.01, *** for p ≤ 0.001.

# indicates that the comparisons were made between patients meeting primary new onset AF vs. those that did not.
